# Nitric oxide-induced lipophagic defects contribute to testosterone deficiency in rats with spinal cord injury

**DOI:** 10.3389/fendo.2024.1360499

**Published:** 2024-02-22

**Authors:** Yuge Zhuang, Wenyuan Liu, Feilong Chen, Minyu Xie, Hanbin Zhang, Zicong Huang, Xiaoyuan Zhang, Jinsheng Liu, Ke Ma, Hongrui Feng, Shipeng Ruan, Jing He, Wansong Zhang, Feng Zou, Xiangjin Kang, Yong Fan, Guofei Zhang, Zhenguo Chen

**Affiliations:** ^1^Guangdong Provincial Key Laboratory of Construction and Detection in Tissue Engineering, Department of Cell Biology, School of Basic Medical Sciences, Southern Medical University, Guangzhou, Guangdong, China; ^2^Department of Pathology, Panyu Maternal and Child Care Service Centre of Guangzhou, Guangzhou, Guangdong, China; ^3^Department of Obstetrics and Gynecology, Key Laboratory for Major Obstetric Diseases of Guangdong Province, Key Laboratory of Reproduction and Genetics of Guangdong Higher Education Institutes, The Third Affiliated Hospital of Guangzhou Medical University, Guangzhou, Guangdong, China; ^4^Department of Urology, The Seventh Affiliated Hospital, Southern Medical University, Foshan, Guangdong, China

**Keywords:** spinal cord injury, Leydig cells, nitric oxide, lipophagy, testosterone

## Abstract

**Introduction:**

Males with acute spinal cord injury (SCI) frequently exhibit testosterone deficiency and reproductive dysfunction. While such incidence rates are high in chronic patients, the underlying mechanisms remain elusive.

**Methods and results:**

Herein, we generated a rat SCI model, which recapitulated complications in human males, including low testosterone levels and spermatogenic disorders. Proteomics analyses showed that the differentially expressed proteins were mostly enriched in lipid metabolism and steroid metabolism and biosynthesis. In SCI rats, we observed that testicular nitric oxide (NO) levels were elevated and lipid droplet-autophagosome co-localization in testicular interstitial cells was decreased. We hypothesized that NO impaired lipophagy in Leydig cells (LCs) to disrupt testosterone biosynthesis and spermatogenesis. As postulated, exogenous NO donor (S-nitroso-N-acetylpenicillamine (SNAP)) treatment markedly raised NO levels and disturbed lipophagy via the AMPK/mTOR/ULK1 pathway, and ultimately impaired testosterone production in mouse LCs. However, such alterations were not fully observed when cells were treated with an endogenous NO donor (L-arginine), suggesting that mouse LCs were devoid of an endogenous NO-production system. Alternatively, activated (M1) macrophages were predominant NO sources, as inducible NO synthase inhibition attenuated lipophagic defects and testosterone insufficiency in LCs in a macrophage-LC co-culture system. In scavenging NO (2-4-carboxyphenyl-4,4,5,5-tetramethylimidazoline-1-oxyl-3-oxide (CPTIO)) we effectively restored lipophagy and testosterone levels both *in vitro* and *in vivo*, and importantly, spermatogenesis *in vivo*. Autophagy activation by LYN-1604 also promoted lipid degradation and testosterone synthesis.

**Discussion:**

In summary, we showed that NO-disrupted-lipophagy caused testosterone deficiency following SCI, and NO clearance or autophagy activation could be effective in preventing reproductive dysfunction in males with SCI.

## Introduction

Globally, spinal cord injury (SCI) affects 40–80 individuals/million every year (1). Thanks to recent medical developments, the overall 10-year survival for SCI is over 80% (2). Therefore, the long-term life quality of surviving patients is an important medical issue. Among SCI complications after injury, reproductive dysfunction is an increasingly concerning issue. While the majority of females with SCI can reproduce (3), in contrast, the overwhelming majority of males cannot reproduce without medical intervention (4). Male reproductive complications include: sexual dysfunction (erectile and/or ejaculatory dysfunction), fertility problems (impaired spermatogenesis and/or poor sperm quality), and systemic disorders (genitourinary infection and endocrine imbalance) (4–6). Testosterone deficiency, defined as total serum testosterone < 11.3 nmol/L, is among the potential causes of these complications, with its specific impact varying due to individual differences (7, 8). High testosterone deficiency rates of up to 83% are recorded in males with acute SCI (< 4 months after injury), with incidence being still high in chronic patients (> 2 years after injury) (9, 10). However, the etiology underlying testosterone deficiency in males with chronic SCI remains unclear.

Leydig cells (LCs) are the main testosterone production sites, are tightly regulated by the pituitary-hypothalamic-gonad axis, and are directly stimulated by luteinizing hormone (LH) (11). However, a moderate proportion of SCI males have low serum testosterone levels but higher LH levels (12, 13), indicating that the pituitary-hypothalamic-gonad axis is intact, but elevated LH levels mostly arise due to negative feedback responses. Thus, impairments have likely occurred in LC paracrine pathways or the local microenvironment. Indeed, inflammatory cells, particularly macrophages, are increasingly activated at ~24 h following SCI, along with elevated inflammatory factors (14–17), which induce oxidative stress and alter the metabolic microenvironment (18–20). Nitric oxide (NO) is an important member of reactive oxygen species. Although physiological levels are required for testis function (21–24), high NO concentrations can inhibit LC steroidogenesis; mechanisms inhibiting the cytochrome P450 enzyme family (25, 26) or depressing NO/cyclic guanosine monophosphatec (cGMP) signaling to impair ATP-evoked Ca^2+^ currents (27, 28) have been suggested. NO is generated by oxidizing L-arginine to L-citrulline via NO synthases (NOSs), which constitute three isoforms: neuronal NOS (nNOS or NOS1), inducible (iNOS or NOS2), and endothelial (eNOS or NOS3) (29). Studies have reported that rat LCs do not express iNOS or nNOS, while only marginal eNOS quantities are detected by DNA array (20). However, other studies reported that rat LCs expressed iNOS and nNOS by immunohistochemistry and immunocytochemistry (24, 30). Human LCs express nNOS and eNOS (31, 32). In mice, nNOS is strongly localized to LCs by immunohistochemistry (33). However, the ability of mouse LCs to produce NO has not been defined. The prevailing view is that NO is predominantly produced by iNOS in activated macrophages following SCI. Therefore, variations occur among species in terms of NOS isoform expression, and it remains unclear if an endogenous NO-producing system exists in mouse LCs.

Autophagy is highly implicated in testosterone synthesis. Young rat LCs demonstrate high autophagy levels and testosterone synthesis, which largely decline as animals age (34). Inhibiting autophagy in rat LCs decreases free cholesterol and testosterone levels (35). Autophagy regulates testosterone synthesis by degrading the negative regulator of low-density lipoprotein receptor to facilitate cholesterol uptake in LCs (36). Also, m6A mRNA methylation can regulate testosterone synthesis via modulated autophagy in LCs (37). The longevity gene *Sirt1* also regulates testosterone biosynthesis in LCs by modulating autophagy (38). Importantly, NO donors also inhibit autophagy, primarily via dependent JNK1/Bcl-2/Beclin and IKK/AMPK/mTORC1 pathways to disrupt Beclin1/Vps34 associations (39).

In this study, we investigated NO and testosterone production and lipophagy activity in the rat model with SCI, and theorized if NO impaired testosterone synthesis by inhibiting lipophagy in LCs to disrupt reproductive capability. We showed that mouse LCs expressed very low NOS isoforms. Treating primary mouse LCs with an exogenous, but not endogenous NO donor, impaired lipophagy and testosterone secretion. NO clearance or autophagy activation restored lipophagy and testosterone synthesis. NOS inhibition also resumed lipophagy and testosterone production in LCs in a macrophage-LC co-culture system.

## Materials and methods

### Animals

Male Sprague-Dawley rats and C57BL/6J mice came from the Animal Center of the Southern Medical University. Animal studies were approved by the Southern Medical University Committee on the Use and Care of Animals and were performed following Committee guidelines and regulations.

### Rat subacute incomplete thoracic SCI models

Adult male Sprague-Dawley rats (250–300 g) were used to construct subacute incomplete SCI models at the thoracic level, as previously described (40). Briefly, rats were acclimatized in the housing facility for at least 3 days prior to experimentation. Animals were randomly grouped and intraperitoneally anesthetized with 1% pentobarbital sodium (25 mg/kg). In the sham group, the spinal cord at the thoracic level was exposed after T9–T11 laminectomy, and then sutured. In the SCI group, a spinal cord compression injury was generated using an aneurysm clip (Sugita, Japan) with a clamping force of 60 g at T10 for 1 min. After surgery, rats were intramuscularly injected with penicillin (50 000 units/kg/day) for 3 days. Manual urination and nursing were performed twice daily to prevent complications. The Basso-Beattie-Bresnahan (BBB) locomotor rating scale was implemented at 24 h after surgery (41), and rats with scores < 3 were considered successful SCI candidates for further analyses. All rats were euthanized on the seventh day after BBB scoring for following experiments.

### Inhibitor treatments

For *in vivo* treatments and after BBB scoring confirmed SCI, SCI rats were intraperitoneally injected with the NO scavenger, 2-4-carboxyphenyl-4,4,5,5-tetramethylimidazoline-1-oxyl-3-oxide (CPTIO, 20 mg/kg/day; C221, Sigma, Shanghai, China) (42). Two strategies were used: 1) Injections commenced immediately following scoring and lasted for 7 days - CPTIO-7d group and 2) Injections commenced at day 3 after scoring and lasted for 4 days – CPTIO-4d group. For the NOS inhibitor, SCI rats were intraperitoneally injected with NG-Nitroarginine methyl ester (L-NAME) hydrochloride (100 mg/kg/day; A7088, APExBIO, Shanghai, China) for 7 consecutive days. Control SCI rats were injected with saline. All rats were euthanized on the seventh day. For *in vitro* treatments, a working concentration of 150 μM CPTIO or 1 mM L-NAME in culture medium was used.

### LC isolation and primary culture

LCs were isolated as previously described (43). Briefly, adult C57BL/6J mice were euthanized by cervical dislocation and testes aseptically removed. The tunica albuginea was carefully removed and seminiferous tubules and interstitial cells were dispersed by treating decapsulated testes with collagenase IV (1 mg/mL; C5138, Sigma) in DMEM/F12 plus 5% fetal bovine serum (FBS; 164210, Procell, Wuhan, China) in a shaking water bath at 37°C for 5 min. After incubation, cold DMEM/F12 was added to terminate collagenase IV activity. Seminiferous tubules were separated from interstitial cells via gravity sedimentation. Crude interstitial cells were collected by centrifugation at 300×*g* for 5 min and resuspended in 2 mL DMEM/F12 plus 5% FBS after washing three times in phosphate buffered saline (PBS, pH 7.4). To obtain purified LCs, resuspended cells were loaded onto a four-layer discontinuous Percoll gradient (17-0891-02, cytiva, Sweden) consisting of 5%, 30%, 58%, and 70% Percoll dissolved in Hanks’ balanced salt solution (HBSS) and centrifuged at 800×*g* at 4°C for 30 min. Highly purified LCs were distributed in the 30%–58% Percoll fraction. LCs were then carefully collected, washed in PBS, and centrifuged at 300×*g* for 5 min. LC purity was > 95% as determined by 3β-hydroxysteroid dehydrogenase 2 (LCs marker) immunofluorescence. Purified LCs were cultured in DMEM/F12 plus 10% FBS, 100 U/mL penicillin, and 100 µg/mL streptomycin at 37°C in a 5% CO_2_ atmosphere.

### Peritoneal macrophages isolation and culture

Peritoneal macrophages were isolated as described previously (26). Briefly, adult C57BL/6J mice were intraperitoneally injected with 4% sodium thioglycolate (3 mL). After 3 days, mice were euthanized by cervical dislocation and soaked in 75% ethanol for 5 min. Using sterile procedures, cells were obtained by peritoneal lavage using calcium- and magnesium-free HBSS. Cell suspensions were centrifuged at 300×*g* for 5 min at 4°C and supernatants discarded. Then, 5 mL DMEM plus 10% FBS, 100 U/mL penicillin, and 100 µg/mL streptomycin were added to resuspend cell pellets, and cell counting and culturing performed. After 6 h, culture supernatants were discarded, cells washed twice in PBS to remove non-adherent cells, and complete medium added to continue culturing. To stimulate NO production, 100 ng/mL lipopolysaccharide (LPS; L4391, Sigma) and 50 ng/mL interferon-γ (IFN-γ; AF-315-05, PeproTech) were added to induce PMs to M1 phenotypes. For PM and LC co-culture, transwell plates were used; PMs were cultured in lower plates while LCs were cultured in inserts.

### Tissue collection and histological analysis

Following euthanasia, the testes and epididymides were removed and weighed, fixed in modified Davidson fixative, and processed in paraffin according to standard methods. At least three sections from each testis and epididymis (3 μm, taken 100 μm apart) were stained with hematoxylin and eosin (H&E) for regular histological examination. Photoshop CS6 Extended (13.0.1; Adobe) was used to analysis the thickness of seminiferous epithelium, diameter of seminiferous lumen and diameter of seminiferous tubule, and area of intestitium.

### Sperm counting

One cauda epididymis was removed from each rat and minced in 1 mL of M2 medium (M7167, sigma) containing 3% bovine serum albumin at 37°C for 20 min to allow the sperm to be released into the medium. The total number of sperm in the final suspension was counted with a hemocytometer.

### Immunofluorescence and confocal microscopy

Immunofluorescence (IF) was performed according to standard procedure, using the primary antibodies summarized in **Supplementary Table 1** and Alexa-Fluor-488-labeled or Alexa-Fluor-594-labeled secondary antibodies (Jackson Immunoresearch, West Grove, PA, USA). 4, 6-diamidino-2-phenylindole (DAPI) was used to visualize nuclei. Immunofluorescent images were obtained and staining intensity was analyzed using a FluoView FV1000 confocal microscope (Olympus, Tokyo, Japan). The fluorescence intensity was quantified using the Image J software (1.53t, National Institutes of Health, USA).

For BODIPY staining, 20 µm frozen sections or primary LC cells were fixed in 4% paraformaldehyde for 15 min, rinsed with PBS, and subsequently incubated in 1 µg/ml BODIPY (D3922, Invitrogen, Carlsbad, CA, USA)-PBS solution at 37 °C for 15 min in dark, alone or followed by immunofluorescent staining with indicated antibodies.

For LysoTracker staining, 50 nM LysoTracker reagents (lysosome fluorescent probe, C1046, Beyotime Biotechnology) were added to LCs culture medium and incubated for 30 min at 37°C. After washing by PBS, fresh culture medium was added and subsequently observed under a confocal microscope.

Cell apoptosis was evaluated by the terminal deoxynucleotidyltransferasemediated dUTP nick end labeling (TUNEL) assay for *in situ* visualization of DNA fragmentation using a commercial kit (DeadEndTM Fluorometric TUNEL System, G3250, Promega, Madison, WI, USA). At least three sections from each testis (3 μm, taken 100 μm apart) were stained. Signals were captured by a confocal microscope. TUNEL-positive germ cells were quantified in each tissue section by counting the number of TUNEL-positive cells in each seminiferous tubule.

### Immunoblotting

Following euthanasia, rat testes were immediately removed, triturated, and lysed on ice with RIPA lysis buffer (P0013B, Beyotime Biotechnology) supplemented with protease and phosphatase inhibitor cocktail (P1046, Beyotime Biotechnology). After centrifugation at 12 000 ×*g* at 4 °C, the supernatant was collected and the protein concentration was quantified by a BCA kit (C503021, Sangon Biotech, Shanghai, China), and finally boiled in sodium dodecyl sulfate (SDS) loading buffer. Thirty microgram of protein was then subjected to 6–12% SDS-polyacrylamide gel electrophoresis and electrotransferred to nitrocellulose membranes (10600001, GE Healthcare Life Sciences). The membranes were then blocked in 5% nonfat dry milk for 1 h at room temperature, washed, and incubated with primary antibodies summarized in **Supplementary Table 1** at 4 °C overnight. The membranes were further washed, incubated with horseradish peroxidase-conjugated secondary antibody (Jackson Immunoresearch) for 1 h at room temperature, washed again, and finally visualized using an enhanced chemiluminescence kit (NEL105001EA, PerkinElmer, Waltham, MA, USA). Quantification was performed by measuring the gray value of bands using the Image J software (1.53t, National Institutes of Health, USA).

### Quantitative reverse-transcription polymerase chain reaction

Total RNA was purified using the Trizol Reagent (Invitrogen), and processed to cDNA using the Hifair™ II 1^st^ Strand cDNA Synthesis Kit, followed by amplification and quantification using the Hieff^®^ qPCR SYBR Green Master Mix (all from YEASEN Biotech, Shanghai, China) with a StepOne Plus Real-Time PCR System (Applied Biosystems, Waltham, MA, USA). *Gapdh* was used as the endogenous control transcript. Three technical replicates were applied for each transcript. The 2-^ΔΔCt^ method was used to calculate the fold changes in gene expression. The primer sequences are summarized in **Supplementary Table 2**.

### Oil Red O staining

20 µm frozen testicular sections were fixed in 4% paraformaldehyde for 15 min and washed three times with PBS. Sections were then incubated in 60% (vol/vol) isopropanol for 5 min and air-dried. Sections were immersed in Oil Red O solution (O0625; Sigma-Aldrich; Oil Red O saturated solution in 3:2 isopropanol/water) for 15 min. Sections were then rinsed in 60% isopropanol for 5-10 s to remove background staining. After rinsing with tap water, sections were counterstained with hematoxylin and placed in 1:1 glycerol/PBS for further analysis. The images were observed and acquired though a light microscope (Carl Zeiss, Germany). Image Pro Plus 6.0 software was used to calculate the ratio of oil red O staining lipid droplet area to total area.

### Enzyme-linked immunosorbent assay

Testosterone level was measured by an enzyme-linked immunosorbent assay (ELISA) kit (ELK1332, ELK Biotechnology, Wuhan, China; detection range: 62.5-4000 pg/mL). For testis, values were normalized to tissue weights and for Leydig cells were to protein concentration. Rat serum luteinizing hormone (LH) level was measured by an ELISA kit (ELK2367, ELK Biotechnology, detection range: 0.47-30 mIU/mL).

### Free cholesterol determination

The concentration of free cholesterol in LCs was measured using an Amplex^®^ Red Cholesterol Assay Kit (A12216, Invitrogen, Detection Range: 200 nM–20 μM). In brief, the supernatant of LC lysates was added to a working solution of 300 μM Amplex^®^ Red reagent containing 2 U/mL horseradish peroxidase, 2 U/mL cholesterol oxidase, and 0.2 U/mL cholesterol esterase and incubated for 30 min at 37°C in dark. Fluorescence was measured in a microplate reader using excitation in the range of 530–560 nm and emission detection at 590 nm. Free cholesterol levels were calculated based on the standard curve and normalized to protein concentration.

### NO probing and measurement

NO level was measured by a NO assay kit (BC1475, Solarbio, Beijing, China, Detection Range: 0.00078-0.25 μmol/mL). The cellular NO in LCs was monitored using the fluorescent NO probe DAF-FM DA (S0019, Beyotime Biotechnology). LCs were cultured in medium with 50 μM DAF-FM DA for 30 min at 37°C. The fluorescence was visualized by a confocal microscope or measured by a FACScan flow cytometer (BD Immunocytometry Systems).

### Proteomics analysis by TMT labeling

At least three testis samples from SCI and sham groups were taken for Tandem mass tag (TMT)-based proteomic analysis. The primary experimental procedures performed by Applied Protein Technology (Shanghai, China) include protein preparation, trypsin digestion, TMT labeling, HPLC fractionation and LC-MS/MS analysis. The MS raw data for each sample were searched using the MASCOT engine (Matrix Science, London, UK; version 2.2) embedded into Proteome Discoverer 1.4 software for identification and quantitation analysis.

### Bioinformatic analysis

#### Cluster analysis

Cluster 3.0 (http://bonsai.hgc.jp/~mdehoon/software/cluster/software.htm) and Java Treeview software (http://jtreeview.sourceforge.net) were used to performing hierarchical clustering analysis. Euclidean distance algorithm for similarity measure and average linkage clustering algorithm (clustering uses the centroids of the observations) for clustering were selected when performing hierarchical clustering. A heat map was often presented as a visual aid in addition to the dendrogram.

### GO annotation

The protein sequences of the selected differentially expressed proteins were locally searched using the NCBI BLAST+ client software (ncbi-blast-2.2.28+-win32.exe) and InterProScan to find homologue sequences, then gene ontology (GO) terms were mapped and sequences were annotated using the software program Blast2GO. The GO annotation results were plotted by R scripts.

### KEGG annotation

Following annotation steps, the studied proteins were blasted against the online Kyoto Encyclopedia of Genes and Genomes (KEGG) database (https://www.genome.jp/kegg/) to retrieve their KEGG orthology identifications and were subsequently mapped to pathways in KEGG.

### Enrichment analysis

Enrichment analysis were applied based on the Fisher’ exact test, considering the whole quantified proteins as background dataset. Benjamini-Hochberg correction for multiple testing was further applied to adjust derived p-values. And only functional categories and pathways with p-values under a threshold of 0.05 were considered as significant.

### Statistical analyses

All studies were performed in triplicate. Data were presented as the mean ± standard deviation, and differences between groups analyzed using *t*-tests for two groups, or one-way analysis of variance for more than two groups, followed by the Dunnett test for between-group differences in SPSS Ver. 13.0 (SPSS, Chicago, IL, USA). A *P<*0.05 value was considered statistically significant.

## Results

### SCI rats exhibit spermatogenic defects and testosterone deficiency

**Figure 1:**
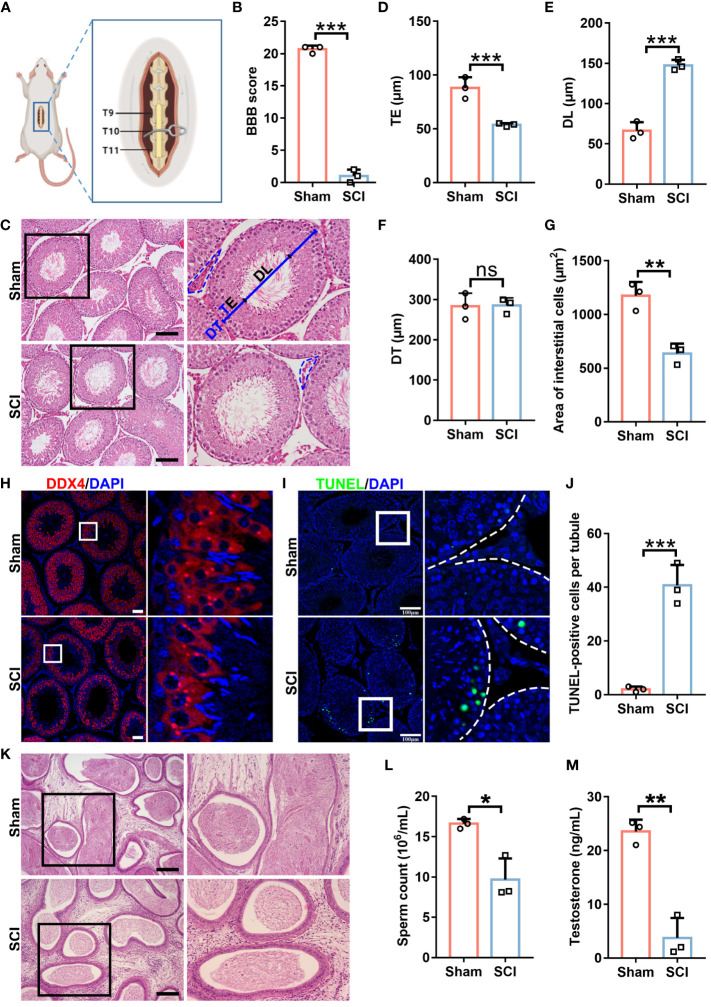
SCI rats exhibit spermatogenic defects and testosterone deficiency. **(A)** Rat model schematic showing subacute incomplete thoracic SCI. **(B)** SCI and sham rat BBB scores. **(C)** Testes histomorphology (H&E staining) in SCI and sham rats. Right panels show magnified views of black boxes. TE, thickness of seminiferous epithelium. DL, diameter of seminiferous lumen. DT, diameter of seminiferous tubule. Black dash lines show the interstitium. **(D)** TE quantification. **(E)** DL quantification. **(F)** DT quantification. **(G)** Quantification of interstitial cell areas. **(H)** DDX4 (germ cell marker) immunofluorescence (red) in testicular sections. Nuclei were stained with DAPI (blue). Views in white boxes are magnified. **(I)** Apoptosis analyses in testicular sections using TUNEL assays. Green fluorescence indicates apoptotic cells. Views in white boxes are magnified. **(J)** TUNEL signal quantification in **(I)**. **(K)** Epididymis histomorphology by H&E staining. Right panels show magnified views of black boxes. **(L)** Sperm released from cauda epididymides. **(M)** Testicular testosterone levels by ELISA assay. Scale bar = 100 μm for **(C, I, K)**, 50 μm for **(H)**. Bars indicate mean values ± standard deviation. *n* = 3 mice for each group and all tubules in each testis section were analyzed, ns = no significance; * *P* < 0.05; ** *P* < 0.01; and *** *P* < 0.001. Data were collected on day 7 after BBB scoring.

We generated a rat SCI model (**Figure 1A**). Basso-Beattie-Bresnahan (BBB) scoring showed that hindlimb movements in injured rats were very subtle or had disappeared, indicating successful model generation (**Figure 1B**). In SCI rats, we observed slightly reduced body weights, while testis weights remained steady, therefore slight increases in testis/body weight ratios were recorded (**Supplementary Figures 1A–C**). Further histomorphological examinations showed that when compared with sham rats, seminiferous tubules in SCI rats had thinner germ cell layers with a larger tubular lumen, but a comparable tubule diameter (**Figures 1C–F**). Interstitial cells in SCI rats were severely degenerated (**Figure 1G**). These observations suggested that spermatogenic defects had arisen in SCI rats. Furthermore, defects were confirmed by the reduced expression of the germ cell marker DDX4 (**Figure 1H**), while increased germ cell loss was identified by TUNEL staining (**Figures 1I, J**). Concordantly, epididymides in SCI rats had significantly lower sperm numbers (**Figures 1K, L**). We excluded injury to epididymides as causing this sperm reduction (**Figure 1K** and **Supplementary Figure 1D**). Additionally, we detected a sharp decrease in testicular testosterone levels in SCI rats (**Figure 1M**), which explained spermatogenic disorders. The serum luteinizing hormone (LH) level in the SCI rats was notably raised, and the mRNA level of *Lhcgr*, a receptor for LH, was also elevated (**Supplementary Figures 1E, F**), indicating that the pituitary gonadotropin-LH pathway is functional and the raise of LH is probably a feed-back compensation. The testosterone decline in SCI rats likely due to impairment in testosterone synthesis within testis. Overall, our rat SCI model exhibited spermatogenic defects and testosterone insufficiency, which largely recapitulated conditions in SCI human males.

### Proteomics reveals disrupted lipid metabolism and steroid biosynthesis in the testes of SCI rats

**Figure 2:**
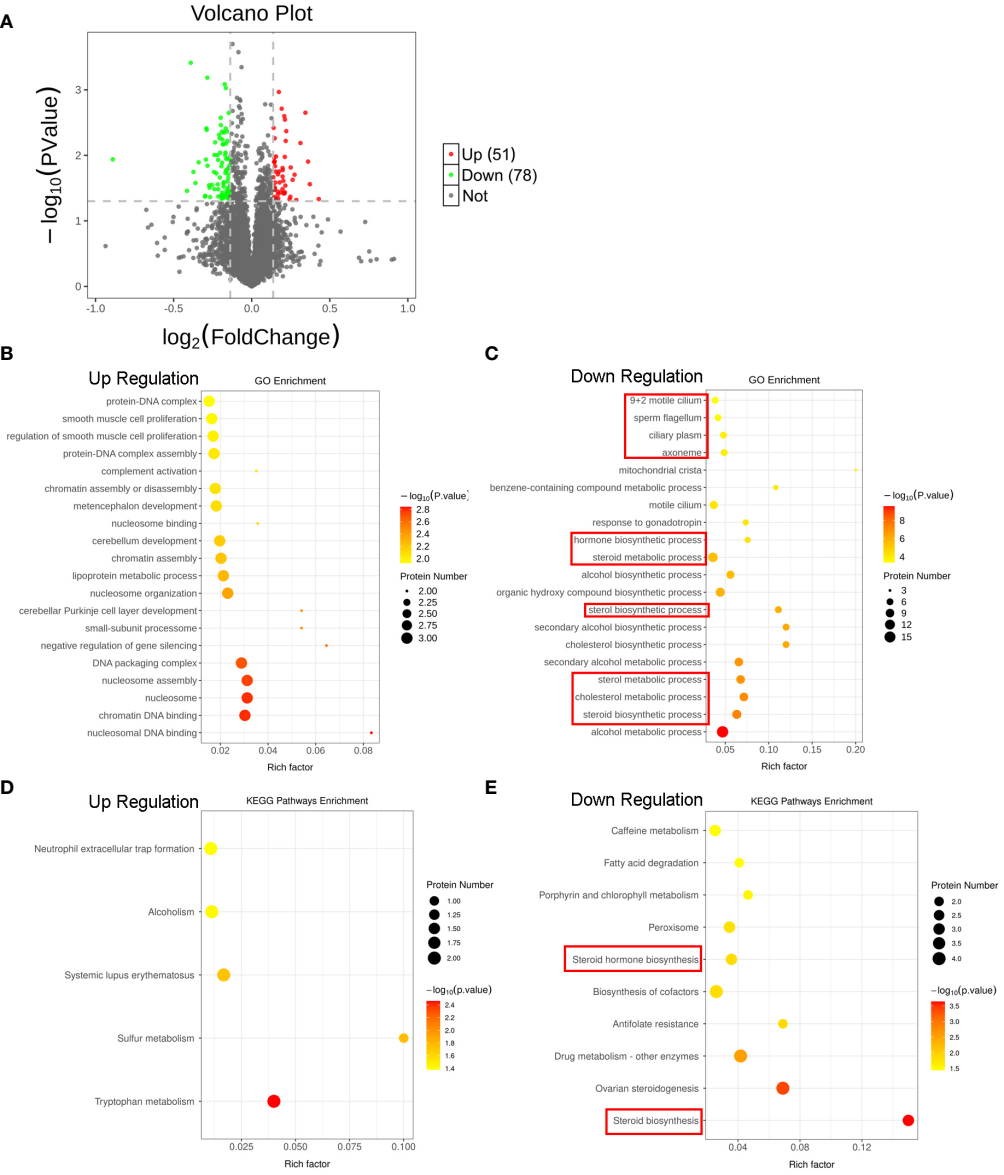
Proteomics reveals SCI disturbs lipid metabolism and steroid biosynthesis in rat testes. **(A)** Volcano plot of the DEPs. Red indicates the upregulated DEPs, and green indicates downregulated DEPs in the testes of SCI rats. **(B, C)** Top 20 up- and down-regulated GO processes associated with DEPs. **(D, E)** Significant KEGG pathways associated with DEPs.

To unveil the global molecular changes underlying the SCI-induced testicular injury, we performed proteomics analyses by isobaric tandem mass tag (TMT) labeling with the sham and SCI testes. As shown in **Figure 2A** and **Supplementary Figure 2B**, volcano plot and heat map indicated 129 differentially expressed proteins (DEPs) in the SCI rats, of which 51 were upregulated and 78 downregulated. Gene Ontology (GO) enrichment analysis showed that the down-regulated DEPs were mostly enriched in steroid and cholesterol metabolism and hormone biosynthesis, as well as sperm flagella (**Figures 2B, C**). Besides, Kyoto Encyclopedia of Genes and Genomes (KEGG) pathway enrichment analysis showed that the down-regulated DEPs were also significantly enriched in steroid and hormone biosynthesis pathways (**Figures 2D, E**). These data globally suggested that after SCI, steroid metabolism and hormone biosynthesis in testis were disrupted, leading to testosterone deficiency and spermatogenic disorders.

### Autophagic activity is depressed and lipid is accumulated in the SCI testes

**Figure 3:**
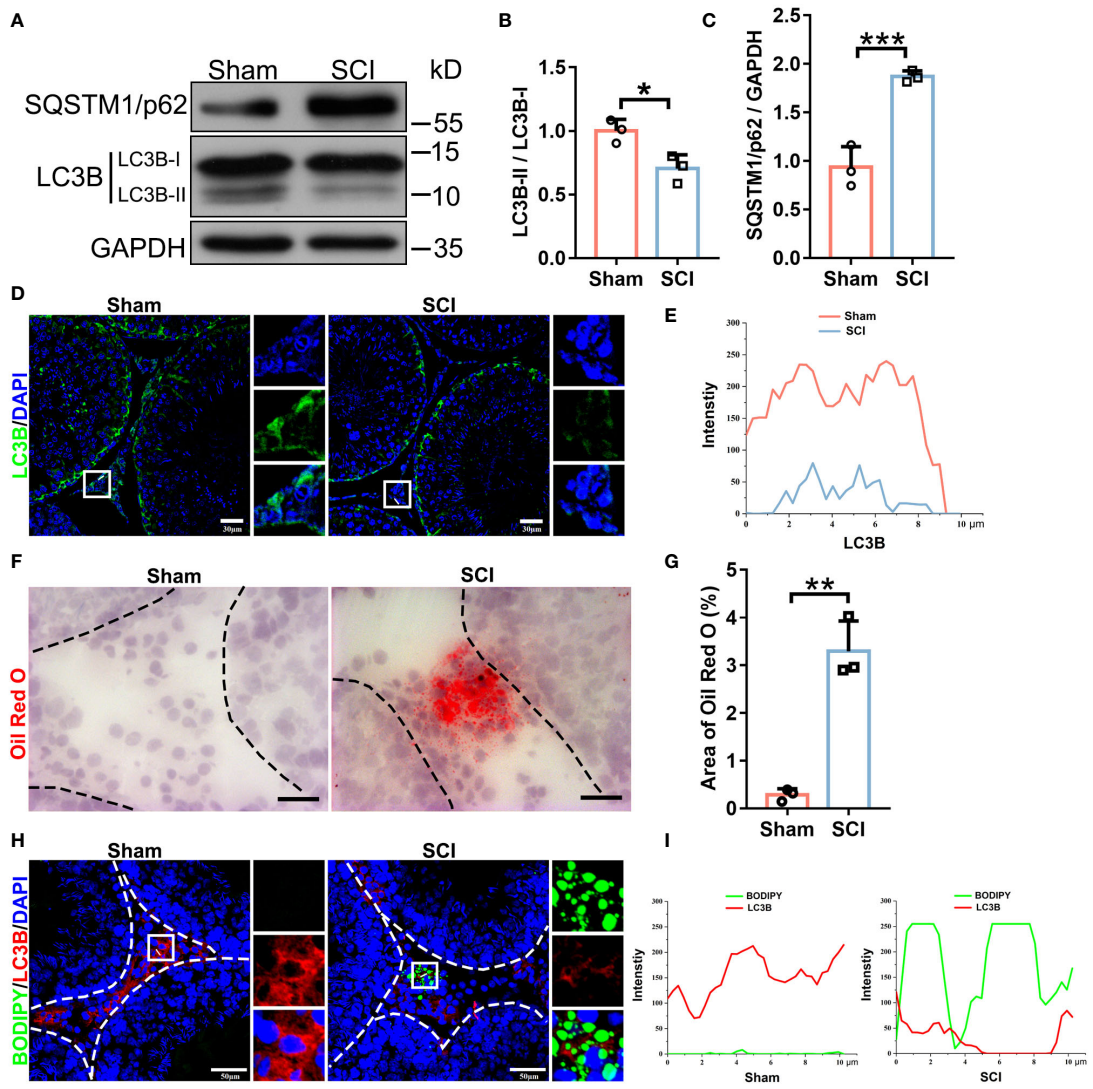
SCI rats exhibit depressed autophagic activity and lipid accumulation in testicular interstitial cells. **(A)** LC3B-I/II and SQSTM1/p62 immunoblotting in sham and SCI testes. **(B, C)** LC3B-II/I and SQSTM1/p62 protein quantification by normalizing levels to GAPDH. **(D)** LC3B immunofluorescence (green) in SCI and sham testicular sections. Nuclei were stained with DAPI (blue). Views in white boxes are magnified. **(E)** LC3B fluorescence quantification on white lines in **(D)**. **(F)** Oil Red O staining (red) showing liquid droplet (LD) abundance in testicular sections. **(G)** Quantification of Oil Red O staining intensity in **(F)**. Data are presented as the proportion of Oil Red O signals in the whole interstitium. **(H)** Lipophagy assays showing BODIPY (green LDs) and LC3B (red autophagosomes) co-fluorescence in testicular sections. **(I)** Co-fluorescence quantification on white lines in **(H)**. Bars indicate mean values ± standard deviation. *n* = 3 mice for each group and all tubules in each testis section were analyzed. * *P* < 0.05; ** *P* < 0.01; and *** *P* < 0.001. Scale bar = 30 μm for **(D)**, 25 μm for **(F)**, and 50 μm for **(H)**.

Autophagy participates in testosterone synthesis. We next tested if SCI impaired autophagy in testes. Immunoblotting showed that SCI testes displayed marked decreases in LC3B isoform protein levels and in LC3B-II to LC3B-I ratios (**Figures 3A, B**), whereas SQSTM1/p62 accumulation indicated blocked autophagy (**Figures 3A, C**). To further analyze these phenomena, we performed LC3B immunofluorescence on testis cross-sections. In sham testes, LC3B positive signals were primarily localized around tubule base membranes and lumen, and also the interstitium. In SCI testes, signals around the base membrane were moderately decreased, while those around the lumen and interstitium had almost vanished (**Figures 3D, E**). Changes in the lumen may have been due to reduced spermatids owing to spermatogenic arrest, as autophagy is required for sperm release. We next focused on lipid deposition and autophagic activity in interstitial cells. Oil red O staining showed that lipid droplets (LDs) were barely detected in sham testes but were distinctly deposited in interstitial cells from SCI rats (**Figures 3F, G**). 4,4-Difluoro-1,3,5,7,8-pentamethyl-4-bora-3a and 4a-diaza-s-indacene (BODIPY - fluorescent dye which labels natural lipids) staining in combination with LC3B immunofluorescence verified increased BODIPY signals, while reduced LC3B signals were observed in SCI testes (**Figures 3H, I**). Combined, these observations suggested that SCI rats had low testosterone levels and impaired spermatogenesis similar to SCI male patients, and their testes showed depressed autophagic activity and lipid accumulation in interstitial cells.

### Lipophagy and testosterone secretion in LCs are inhibited by NO but restored by a NO scavenger

**Figure 4:**
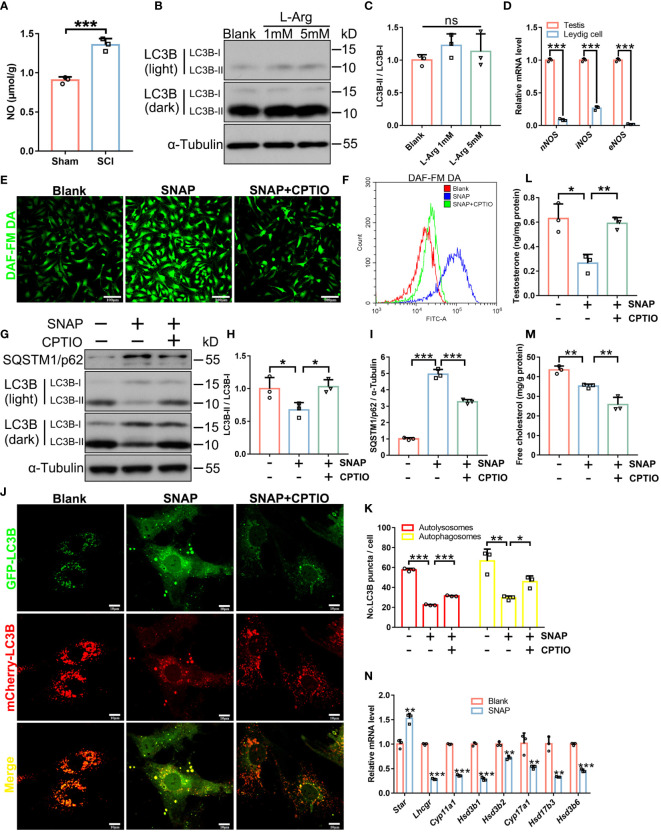
In LCs, NO inhibits autophagy and testosterone secretion which are restored by CPTIO. **(A)** NO levels in SCI and Sham testes. Data are presented as μmol/g protein. **(B)** LC3B-I/II immunoblotting in L-Arginine-treated and control primary LCs. **(C)** LC3B-II/I quantification in **(C)**. **(D)** qRT-PCR analyses of *nNOS*, *iNOS*, and *eNOS* mRNA levels in whole testis extracts and primary LCs. **(E)** DAF-FM DA fluorescence (green) showing cellular NO abundance in control, SNAP-, and SNAP+CPTIO-treated primary LCs. LCs were cultured in normal medium, or in medium plus SNAP (500 μM) alone or combined with CPTIO (150 μM) for 24 h. **(F)** Quantification of DAF-FM DA intensity by flow cytometry in **(E)**. **(G)** LC3B-I/II and SQSTM1/p62 immunoblotting in control, SNAP-, and SNAP+CPTIO-treated LCs. **(H, I)** LC3B-II/I and SQSTM1/p62 quantification in **(G)**. **(J)** Autophagic flux revealed by mCherry-GFP-LC3B dual-fluorescence. LCs were transfected with an adenovirus harboring mCherry-GFP-LC3B for 24 h, followed by SNAP or SNAP+CPTIO treatment for another 24 h and then observed under confocal microscopy. **(K)** Autophagic puncta quantification in **(J)**. Red puncta indicate autolysosome (GFP signals are quenched in low pH environments in lysosomes) and yellow puncta indicate autophagosomes. **(L)** Testosterone concentrations in medium from indicated groups by ELISA. Data are presented as ng/mg protein. **(M)** Cell free cholesterol concentrations in indicated groups. Data are presented as mg/g protein. **(N)** qRT-PCR analyses of *Star*, *Lhcgr*, *Cyp11a1*, *Hsd3b1*, *Hsd3b2*, *Cyp17a1*, *Hsd17b3*, and *Hsd3b6* mRNA levels in control and SNAP-treated LCs. Bars indicate mean values ± standard deviation. *n* = 3, ns = no significance; * *P* < 0.05; ** *P* < 0.01; and *** *P* < 0.001. Scale bar = 100 μm for **(E)** and 50 μm for **(J)**.

NO levels were significantly elevated in SCI rat testes (**Figure 4A**). Therefore, we investigated if NO disrupted autophagy and subsequently impaired steroidogenesis and testosterone synthesis. We used mouse primary LCs (**Supplementary Figure 2B**), tested NO production in LCs, and examined to what extent an endogenous NO donor (L-arginine) produced NO in LCs. L-arginine failed to increase NO levels in LCs (DAF-FM DA staining, **Supplementary Figure 2C**) and barely affected LC3B-I/II protein levels (**Figures 4B, C**), even at 5 mM. Our qRT-PCR analyses showed that iNOS and nNOS genes were very weakly expressed, and eNOS was barely detectable in mouse LCs (**Figure 4D**). Thus, mouse LCs produced low NO levels. In contrast, an exogenous NO donor (S-nitroso-N-acetylpenicillamine (SNAP)) (25) significantly increased NO levels in LCs (**Figures 4E, F**), decreased LC3B-II while increasing LC3B-I levels, and accumulated the autophagic substrate SQSTM1/p62 (**Figures 4G–I**). Consistently, LC3B-GFP^–^mCherry^+^ (red autophagosomes) and LC3B-GFP^+^/mCherry^+^ (yellow autolysosomes) dual-immunofluorescence signals were significantly decreased after SNAP treatment (**Figures 4J, K**), indicating blocked autophagic processes in SNAP-treated LCs. Also, testosterone synthesis was largely disrupted (**Figure 4L**) as cholesterol (**Figure 4M**) and steroidogenesis enzyme levels were decreased after SNAP treatment (**Figure 4N**). Thus, the exogenous NO donor (not the endogenous NO donor) produced NO, inhibited autophagy, and impeded testosterone production in mouse LCs, consistent with *in vivo* observations in SCI rats.

**Figure 5:**
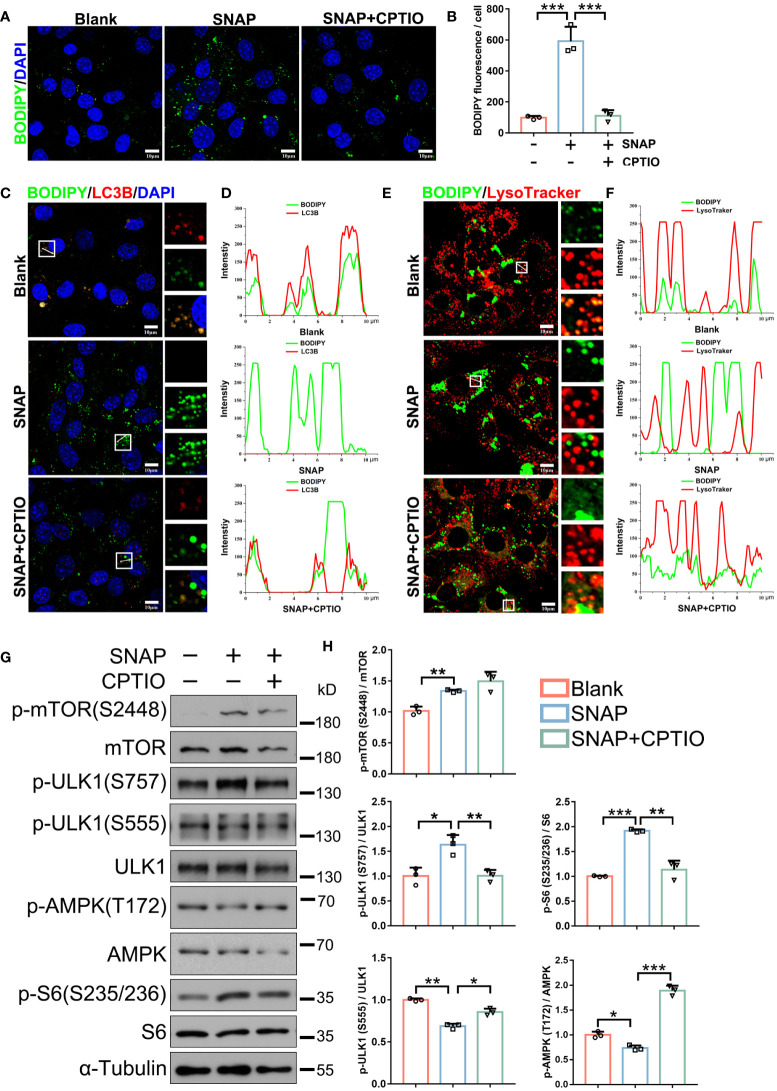
CPTIO restores NO-impaired lipophagy in LCs. **(A)** BODIPY (green) staining shows LD abundance in indicated groups. Nuclei were stained with DAPI (blue). **(B)** BODIPY fluorescence quantification in **(A)**. **(C)** Lipophagy assays showing BODIPY (green, LDs) and LC3B (red autophagosomes) co-fluorescence. Views in white boxes are magnified. **(D)** Co-fluorescence intensity quantification on white lines in **(C)**. **(E)** Lipophagy assays showing BODIPY (green LDs) and LysoTracker (red, lysosomes) co-fluorescence. Views in white boxes are magnified. **(F)** Co-fluorescence intensity quantification on white lines in **(E)**. **(G)** Immunoblotting showing autophagy-associated mTOR, ULK1, and AMPK pathway activity. **(H)** Quantitative analyses of **(G)**. Bars indicate mean values ± standard deviation. *n* = 3, ns = no significance; * *P* < 0.05; ** *P* < 0.01; and *** *P* < 0.001. Scale bar = 10 μm.

As observed in SCI rat testis sections, SNAP also induced lipid accumulation in primary mouse LCs (**Figures 5A, B**). We next monitored NO effects on lipophagy. Double BODIPY/LC3B fluorescence further confirmed decreased LC3B signals while LDs had accumulated in SNAP-treated mouse LCs, and strikingly, BODIPY/LC3B co-localization was almost abolished (**Figures 5C, D**), indicating that SNAP had disrupted LD engulfment into autophagosomes, an early and essential lipophagy step. Autophagosomes are destined to fuse with lysosomes so as to degrade substrates for recycling. Consistently, double BODIPY/Lysotracker fluorescence confirmed reductions in co-localized LDs and lysosomes in SNAP-treated LCs (**Figures 5E, F**), further validating blocked lipophagy processes. The mRNA levels of several critical factors involved in lipid uptake or synthesis were decreased (**Supplementary Figure 3A**), indicating that the observed lipid accumulation was not due to increase in lipid synthesis. To corroborate our evidence, treatment with 2-4-carboxyphenyl-4,4,5,5-tetramethylimidazoline-1-oxyl-3-oxide (CPTIO), a NO scavenger (26), significantly reversed SNAP-induced NO elevation, restored autophagy, especially lipophagy, and therefore promoted testosterone production (**Figures 4E–L**, **5A–F**). Unexpectedly, CTIOP lowered cellular cholesterol levels (**Figure 4M**) and downregulated factors required for lipid uptake (**Supplementary Figure 3B**). These observations require further investigations. However, of note, CPTIO alone did not alter autophagic activity in LCs (**Supplementary Figure 3C**), indicating that CPTIO reversed SNAP-induced lipophagic impairment by specifically clearing NO. Cumulatively, these results demonstrated that NO suppressed lipophagy and testosterone production in mouse LCs, which were effectively rescued by CPTIO, a NO scavenger.

### Autophagy activation resumes NO-blocked lipophagy and testosterone synthesis in LCs

We next determined which autophagy-associated pathways were altered in SNAP-treated LCs. Immunoblotting showed that the mTOR pathway, which inhibits autophagy, was activated as indicated by upregulated p-mTOR (S2448) and the downstream p-S6 protein (S235/S236) (**Figures 5G, H**). ULK1 phosphorylation at S757, which is activated by mTOR to inhibit autophagy, was also upregulated. Both AMPK (T172) and downstream ULK1 (S555) activities, which promote autophagy, were also downregulated (**Figures 5G, H**). Accordingly, SQSTM1/p62 accumulated in cells. Critically, these signaling alterations were reversed by CPTIO (**Figures 5G, H**). When combined, NO inhibited LC autophagy, which upregulated anti-autophagic mTOR and downregulated pro-autophagic AMPK/ULK1.

**Figure 6:**
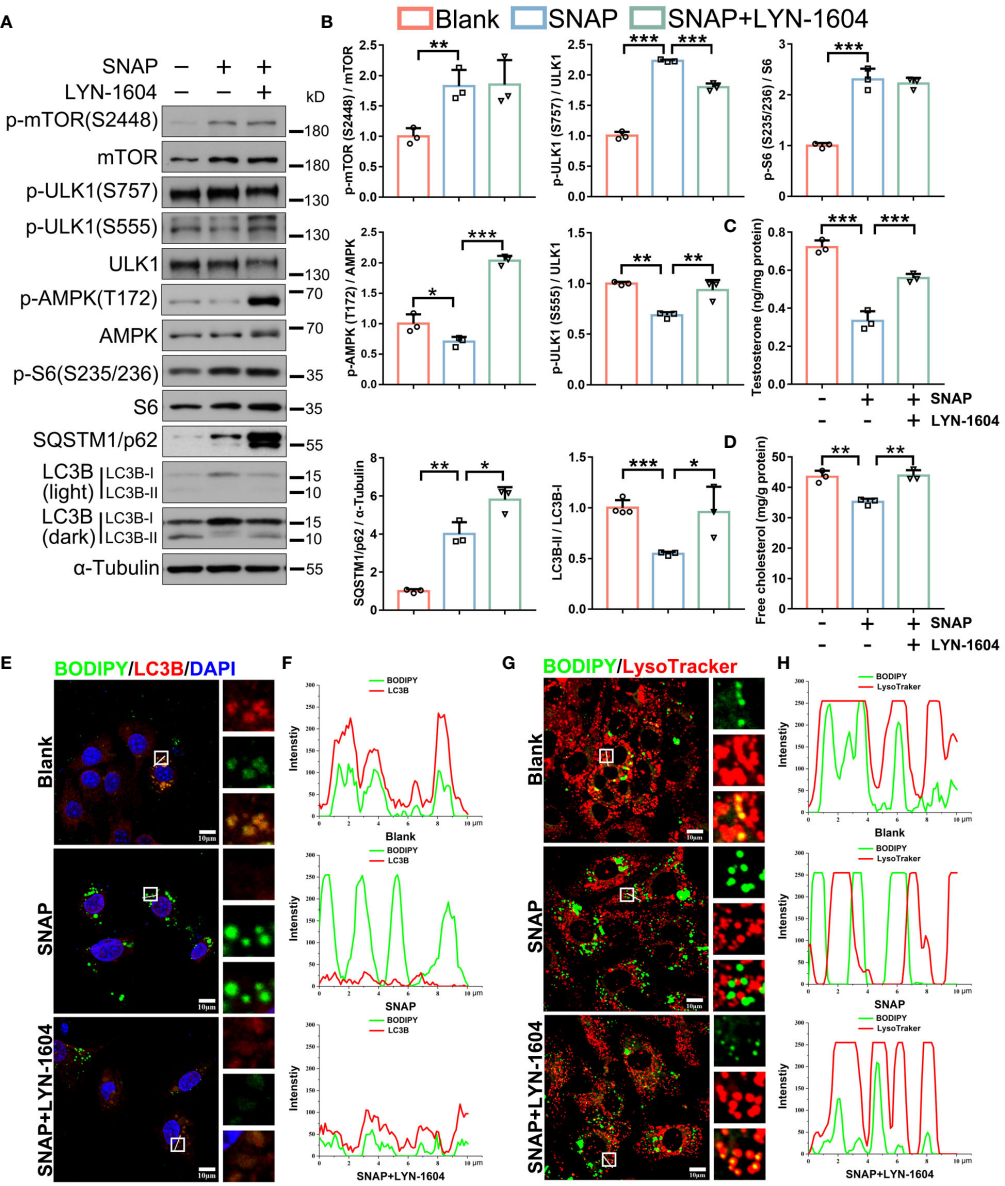
Autophagy activation by LYN-1604 restores NO-depressed lipophagy and testosterone synthesis in LCs. **(A)** Autophagic activity and pathway immunoblotting in control, SNAP-, and SNAP+LYN-1604-treated LCs. LCs were cultured in normal medium, or in medium plus SNAP (500 μM) alone or combined with LYN-1604 (50 μM) for 24 h. **(B)** Protein quantification in **(A)**. **(C)** Testosterone concentrations in medium in indicated groups by ELISA. Data are presented as ng/mg protein. **(D)** Cell free cholesterol levels in indicated groups. Data are presented as mg/g protein. **(E)** Lipophagy assays showing BODIPY (green LDs) and LC3B (red, autophagosomes) co-fluorescence. Views in white boxes are magnified. **(F)** Co-fluorescence intensity quantification on white lines in **(E)**. **(G)** Lipophagy assays showing BODIPY (green LDs) and LysoTracker (red lysosomes) co-fluorescence. Views in white boxes are magnified. **(H)** Co-fluorescence intensity quantification on white lines in **(G)**. Bars indicate mean values ± standard deviation. *n* = 3, ns = no significance; * *P* < 0.05; ** *P* < 0.01; and *** *P* < 0.001. Scale bar = 10 μm.

We next asked if activating upstream autophagy signaling antagonized NO in mouse LCs. LYN-1604 treatment [ULK1 agonist (44)] activated SNAP-depressed AMPK/ULK1 (S555) signaling and autophagic activity (**Figures 6A, B**). Correspondingly, LYN-1604 efficiently restored LDs engulfment into autophagosomes and lysosome delivery (degradation) (**Figures 6E–H**; **Supplementary Figures 4C, D**), and importantly, cholesterol and testosterone levels were restored (**Figures 6C, D**). These results showed that NO inhibited lipophagy and testosterone synthesis in mouse LCs, and that LYN-1604 alleviated these phenotypes by specifically activating upstream autophagic signaling, as LYN-1604 did not alter cellular NO levels in LCs (**Supplementary Figures 4A, B**).

### NO scavenging by CPTIO restores testosterone levels and spermatogenesis in SCI rats

**Figure 7:**
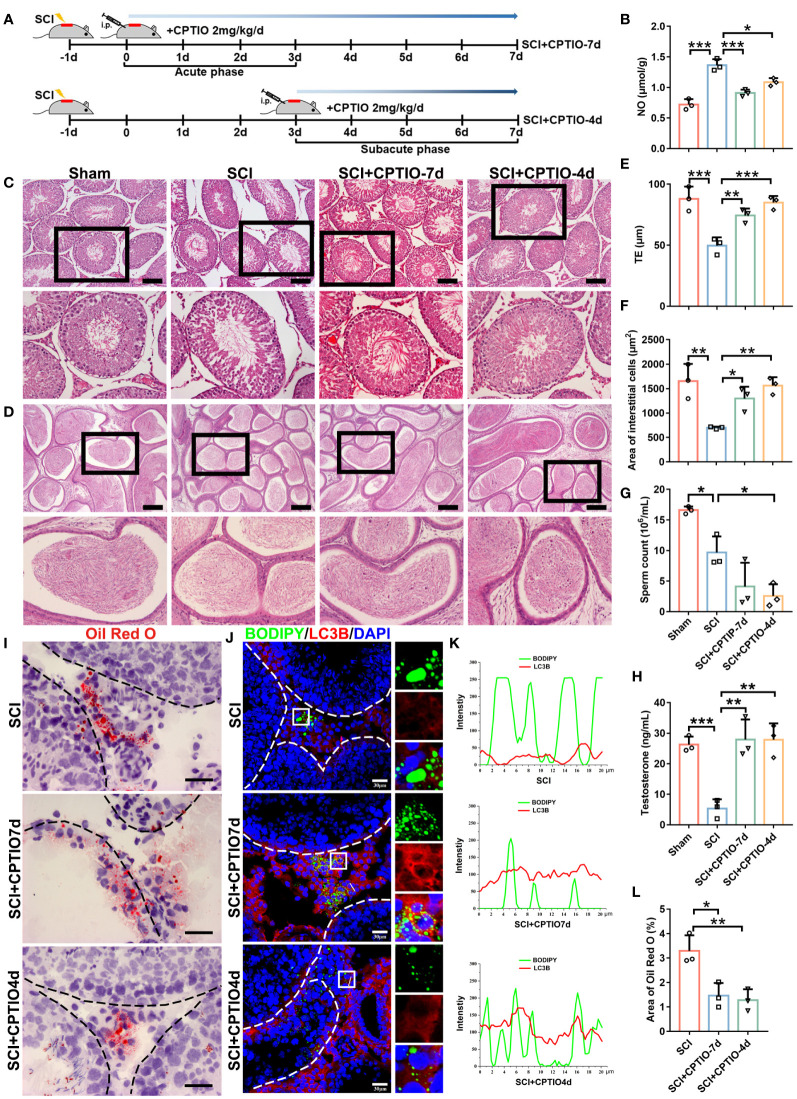
NO scavenging by CPTIO restores testosterone levels and spermatogenesis in SCI rats. **(A)** Schematic showing the *in vivo* CPTIO administration strategy (2 mg/kg/d) in SCI rats. At 24 h after surgery, BBB scoring was performed, which day was designated as d0. SCI+CPTIO-7d group: CPTIO administration began at d0, covered the acute phase (d0–d3), and lasted for 7d; SCI+CPTIO-4d group: CPTIO administration began at d3, covered the subacute phase (d3–d7), and lasted for 4d. **(B)** Testicular NO levels in each group. **(C)** H&E staining showing testicular morphology in indicated groups. Views in black boxes are magnified in the panel below. **(D)** H&E staining showing epididymal morphology in indicated groups. Views in black boxes are magnified in the panel below. **(E)** TE quantification. TE, thickness of seminiferous epithelium. **(F)** Quantification of interstitial cell areas. **(G)** Sperm released from one cauda epididymis from each rat. **(H)** Testicular testosterone levels by ELISA. **(I)** Lipid deposition by Oil Red O staining (red). **(J)** Quantification of Oil Red O intensity in **(H)**. Data are presented as the proportion of Oil Red O signals in the whole interstitium. **(K)** Lipophagy assays showing BODIPY (green LDs) and LC3B (red autophagosomes) co-fluorescence. Nuclei were stained with DAPI (blue). Views in white boxes are magnified. **(L)** Co-fluorescence intensity quantification on white lines in **(J)**. Data indicate mean values ± standard deviation. *n* = 3 mice for each group and all tubules in each testis section were analyzed ns = no significance; * *P* < 0.05; ** *P* < 0.01; and *** *P* < 0.001. Scale bar = 100 μm for **(B, C)**, 25 μm for **(H)**, and 30 μm for **(J)**.

We next evaluated *in vivo* CPTIO efficacy in restoring testosterone levels and spermatogenesis. In mice or rats, acute immune responses begin at ~24 h after SCI, with a duration of approximately 3 days (45). Based on this information and using BBB scoring to confirm SCI, we used two CPTIO treatment strategies in SCI rats: 1) treatment began immediately following scoring and lasted for 7 days - CPTIO-7d group; 2) treatment began at day 3 and lasted for 4 days - CPTIO-4d group (**Figure 7A**). We were keen to identify which approach improved testicular function/recovery; i.e., eliminating NO as soon as possible (CPTIO-7d) or allowing NO production during acute immune stages (CPTIO-4d). Both treatments significantly decreased NO levels (**Figure 7B**). H&E staining showed that tubular histomorphology was restored in both groups, with epithelium thickness and interstitial cell areas significantly recovered (**Figures 7C, E, F**). The CPTIO-4d strategy appeared to show better effects when compared with CPTIO-7d. Both CPTIO treatments also enhanced lipophagic activity in interstitial cells, avoided aberrant lipid accumulation (**Figures 7I–L**), and promisingly, restored testosterone levels (**Figure 7H**). Surprisingly, neither CPTIO treatments restored sperm release from epididymides (**Figure 7G**). However, we noted that sperm reservoirs in epididymides were morphologically recovered (**Figure 7D**). Overall, these observations, in combination with *in vitro* data, indicated that NO scavenging by CPTIO attenuated SCI-impaired lipophagy and testicular functions.

### *In vitro* but not *in vivo* treatment with a NOS inhibitor (L-NAME) restores lipophagy and testosterone levels

**Figure 8:**
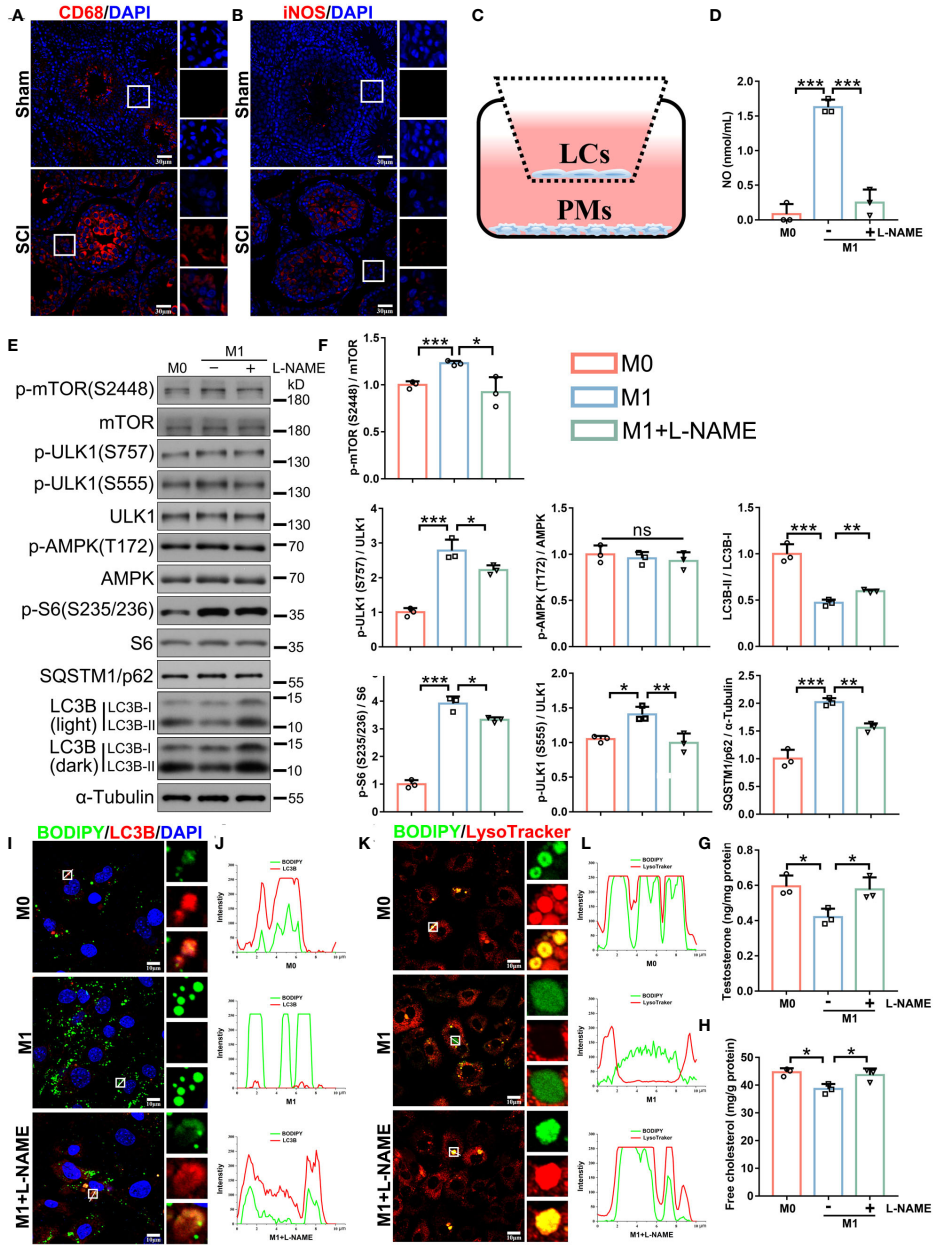
*In vitro* treatment with a NOS inhibitor (L-NAME) restores lipophagy and testosterone levels. **(A, B)** CD68 (red, **A**) or iNOS (red, **B**) immunofluorescence in SCI and sham testes. DAPI indicates nuclei (blue). Views in white boxes are magnified. **(C)** Schematic showing the peritoneal macrophage (PM) and LC co-culturing system. **(D)** NO concentrations in M0 macrophage (inactivated) and M1 macrophage (activated) medium, or M1 medium plus 1 mM L-NAME for 24 h. **(E)** Immunoblotting analyses showing autophagy-associated pathways. **(F)** Data quantification from **(E)**. **(G)** Testosterone levels in LCs by ELISA. **(H)** Free cholesterol levels in LCs. **(I)** Lipophagy assays showing BODIPY (green LDs) and LC3B (red autophagosomes) co-fluorescence. Views in white boxes are magnified. **(J)** Co-fluorescence intensity quantification on white lines in **(I)**. **(K)** Lipophagy assays showing BODIPY (green LDs) and LysoTracker (red lysosomes) co-fluorescence. Views in white boxes are magnified. **(L)** Co-fluorescence intensity quantification on white lines in **(G)**. Data indicate mean values ± standard deviation. *n* = 3, ns = no significance; * *P* < 0.05; ** *P* < 0.01; *** and *P* < 0.001. Scale bar = 30 μm for **(A, B)**. Scale bar = 10 μm for **(I, K)**.

We observed the increased testicular expression of two macrophage markers CD68 and iNOS (**Figures 8A, B**), further suggesting that NO was predominantly produced by macrophages and mediated inflammation-impaired steroidogenesis. We therefore explored if NO clearance, by directly inhibiting NOS, effectively recovered testosterone levels and spermatogenesis *in vivo*. To this end, SCI rats were injected with a NOS inhibitor (L-NAME) (24). Unexpectedly, *in vivo* analyses showed neither restored testosterone levels, nor testis morphology, nor sperm counts (**Supplementary Figure 5**). This was likely due to the general effects of L-NAME in blocking all NOS isoforms in testes. To overcome this issue, we used a co-culture system, with LCs (2 × 10^5^ cells/well) in upper and peritoneal macrophages (PMs, 5 × 10^5^ cells/well) in lower Transwell chambers, to mimic the immune-activated microenvironment *in vivo* (**Figure 8C**). Both cell types had no direct contact and communicated with each other only via signaling. At first, macrophages were induced by lipopolysaccharide (LPS) and interferon-γ (IFN-γ), under which conditions LCs were co-cultured for 24 h (**Supplementary Figure 6A**). Immunoblotting showed that iNOS protein levels were significantly raised after LPS and INF-γ treatments (**Supplementary Figure 6A**), indicating successful M1 transition. We observed decreased LC3B-II/I ratios (**Supplementary Figure 6B**), indicating that M1 macrophage signals had suppressed LCs autophagy. However, L-NAME treatments failed to rescue LC3B-II/I ratios (**Supplementary Figure 6B**). We hypothesized that LPS and IFN-γ probably compromised L-NAME effects. Indeed, LPS and IFN-γ alone, even without M1 macrophages, also decreased LC3B-II/I ratios (**Supplementary Figure 6B**). Therefore, LPS and IFN-γ were supplemented for the first 24 h for M1 induction and then removed, with M1 macrophages and LCs co-cultured in fresh medium for another 24 h (**Figure 8C**). We observed that M1 macrophages alone produced high NO levels (**Figure 8D**), decreased autophagic activity and pathway outputs (**Figures 8E, F**), disturbed lipophagy, induced lipid accumulation (**Figures 8I–L**), and reduced testosterone secretion in LCs (**Figures 8G, H**), all of which were reversed by L-NAME (**Figures 8D–L**). Thus, NO production by M1 macrophages was a critical incentive for lipophagic defects and testosterone deficiency in LCs, and the application of a NOS inhibitor to clear NO appeared to prevent these phenotypes.

## Discussion

NO inhibits steroidogenesis in LCs (20, 25, 26), with several potential mechanisms proposed in the literature. One study showed that NO neither increased cGMP production nor modified cyclic adenosine monophosphate (cAMP) production, but alternatively, directly inhibited the cytochrome P450 enzyme family (25, 26), which catalyzed several critical reactions during testosterone synthesis. In contrast, another study reported that NO critically elevated cGMP levels, which in turn blocked LH-modulated ATP-evoked Ca^2+^ currents required for testosterone synthesis (27, 28). Interestingly, NO inhibited autophagy via complex inhibitory effects on JNK1/Bcl-2/Beclin 1 and IKK/AMPK/TSC2 pathways (39). In mice, autophagy is required for testosterone biosynthesis by facilitating cholesterol uptake in LCs (36), and mouse LCs are rich in lipid droplets. In contrast, rat LCs barely contain lipid droplets. This may indicate the difference between these two species. We reasoned that lipophagy is required for testosterone synthesis in rats, which was impaired by elevated NO. This hypothesis was supported by our findings where SCI rats exhibited testosterone deficiency and high testicular NO levels, while NO clearance by CPTIO restored lipophagy and testosterone synthesis *in vitro* and *in vivo*. Strikingly, CPTIO administration for 4 or 7 days recovered seminiferous tubular morphology and spermatogenesis *in vivo*, although final reproductive outcomes (breeding pups) were not examined. Unexpectedly, we observed that sperm numbers released from epididymides were further decreased following CPTIO treatment. We postulated that systematic CPTIO administration eliminated excessive NO, therefore disrupting essential basal NO functions in sperm release, maturation, and motility (21). Indeed, sperm swimming abilities from epididymides were decreased (data not shown). CPTIO treatments also appeared to block eNOS activity and alter testicular blood flow, which were possibly important in preventing pro-inflammatory cell and factor accumulation (24, 31, 32). In this regard, CPTIO treatment methods, time-points and durations, and doses should be optimized for future potential clinical applications.

Our findings also suggested that NO-impaired autophagy was a key reason for testicular phenotypes in SCI rats, as autophagy activation by LYN-1604 recovered lipophagy and testosterone production. In considering previous reports showing that aged rats exhibit low testosterone levels and autophagic activity in testes (34), we proposed that promoting autophagy in aged LCs could potentially prevent age-related testosterone deficiency, such as late-onset hypogonadotropin, a common condition in aged man and characterized by testosterone insufficiency concomitant with increasing age (46). Additionally, *S*-nitrosylation and target protein inhibition are the primary NO mechanisms mediating signaling transduction (47). We observed AMPK/ULK1 signal suppression, as revealed by reduced AMPK phosphorylation at T172 and ULK at S555. It was also possible that NO-mediated *S*-nitrosylation of AMPK and ULK, alone or combined, inhibited their expression. Moreover, NO-mediated *S*-nitrosylation of ATG4B induced autophagic impairment and neurotoxicity in response to hyperglycemia (48), thus, the *S*-nitrosylation of autophagy pathway members may represent a new autophagic regulatory mechanism under oxidative stress.

Although nNOS was strongly localized to mouse LCs by immunohistochemistry (33), the ability of LCs to produce NO had not been tested. A single-cell sequencing data base for mouse testis development (http://malehealthatlas.cn/) indicated that mouse LCs have very low transcription levels of all three NOS isoforms. In line with this, our qRT-PCR data showed that primary mouse LCs poorly expressed NOS isoforms, and we also showed that an exogenous donor (SNAP) but not an endogenous NO donor (L-arginine) significantly increased NO levels in these cells. The *in vitro* application of L-NAME to suppress NOS levels restored lipophagy and testosterone production in LCs in M1 macrophage-conditioned medium. These results showed that activated macrophages, and not LCs, produced NO following SCI (20, 26). Since NO is an important neurotransmitter and is involved in the histamine-induced inhibition of LC steroidogenesis (49), we hypothesize that NO has critical communication roles in nervous, immune, and endocrine systems within an SCI context. Further investigations on signaling mechanisms underlying NO *S*-nitrosylation toward target proteins will help decipher these functions. Nevertheless, *in vivo* L-NAME treatment failed to recover testicular function. We theorized that short-term/high-dose L-NAME administration (100 mg/kg/d for 7 days) may have induced overwhelming systemic inhibitory effects on all NOS isoforms, producing effects similar to *in vivo* CPTIO treatments which excessively lowered testicular NO levels. Therefore, in future studies, it will be important to examine if a long-term/low-dose CPTIO scheme (e.g., 8 mg/kg/d for 30 days) (50) or a more specific iNOS inhibitor (1400W) (51) can achieve better effects.

## Conclusions

**Figure 9:**
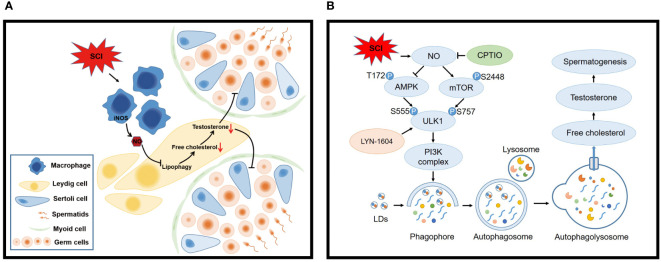
Schematic model showing how macrophage-derived NO disrupts lipophagy and testosterone synthesis in LCs following SCI. **(A)** SCI activates macrophages to release high NO levels which impair lipophagy in LCs, leading to free cholesterol insufficiency and testosterone deficiency, finally disrupting spermatogenesis. **(B)** Mechanistically, NO inhibits lipophagy by interfering with AMPK/mTOR/ULK1 pathways and disturbing autophagy. Consequently, LD engulfment into autophagosomes and lysosome delivery (degradation) are blocked, resulting in cholesterol insufficiency and testosterone deficiency. NO clearance by CPTIO or autophagy activation by LYN-1604 alleviates SCI-induced testicular dysfunction.

In this study, by generating a rat SCI model, we confirmed that SCI disrupted lipid metabolism and steroid biosynthesis, induced testosterone deficiency and spermatogenic disorders, along with increased aberrant NO levels in testes. The NO donor SNAP disrupted lipophagy and testosterone biosynthesis in primary LCs. Mechanistically, NO activated mTOR/ULK1 (S757) and inhibited AMPK/ULK1 (S555) signaling to depress autophagy pathways. NO clearance by CPTIO effectively restored lipophagy and testosterone levels *in vitro* and *in vivo*. Autophagy activation by LYN-1604 also reversed lipid accumulation and testosterone insufficiency. Critically, ours is the first study to demonstrate that mouse LCs are devoid of an L-arginine/NO pathway. NO was predominantly produced by activated macrophages following SCI, and disrupted lipophagy and testosterone production in LCs, which were alleviated by the NOS inhibitor (L-NAME) (**Figure 9**).

## Data availability statement

The original contributions presented in the study are included in the article/**Supplementary Material**. Further inquiries can be directed to the corresponding authors.

## Ethics statement

The animal study was approved by Southern Medical University Committee on the Use and Care of Animals. The study was conducted in accordance with the local legislation and institutional requirements.

## Author contributions

YZ: Conceptualization, Writing – original draft, Writing – review & editing, Data curation, Investigation, Visualization. WL: Investigation, Writing – review & editing, Formal analysis, Project administration, Validation. FC: Formal analysis, Investigation, Validation, Writing – review & editing, Data curation. MX: Writing – review & editing, Methodology. HZ: Methodology, Writing – review & editing. ZH: Methodology, Writing – review & editing. XZ: Writing – review & editing, Validation. JL: Validation, Writing – review & editing. KM: Validation, Writing – review & editing. HF: Writing – review & editing, Investigation. SR: Investigation, Writing – review & editing. JH: Investigation, Writing – review & editing. WZ: Investigation, Writing – review & editing. FZ: Investigation, Writing – review & editing. XK: Writing – review & editing, Funding acquisition, Resources. YF: Writing – review & editing, Funding acquisition, Project administration. GZ: Project administration, Writing – review & editing, Funding acquisition. ZC: Resources, Writing – review & editing, Funding acquisition, Conceptualization, Supervision, Writing – original draft.
