# Supplementary material for: Nitric oxide-induced lipophagic defects contribute to testosterone deficiency in rats with spinal cord injury
Source: Front Endocrinol (Lausanne). 2024 Feb 22;15:1360499. doi: 10.3389/fendo.2024.1360499 (PMC10918589; doi:10.3389/fendo.2024.1360499)
Supplement: Supplementary file 1 [file DataSheet_1.docx]

**Supplementary Materials for**

**Nitric oxide-induced lipophagic defects cause testosterone deficiency after spinal cord injury**

Yuge Zhuang, Wenyuan Liu, Feilong Chen, Minyu Xie, Hanbin Zhang, Zicong Huang, Xiaoyuan Zhang, Jinsheng Liu, Ke Ma, Hongrui Feng, Shipeng Ruan, Jing He, Wansong Zhang, Feng Zou, Xiangjin Kang, Yong Fan, Guofei Zhang, Zhenguo Chen.

Lead correspondence: Zhenguo Chen, e-mail: czg1984@smu.edu.cn.

**This file includes:**

Supplementary Figs. S1 to S6

Supplementary Tables. S1 to S2

**Supplementary Figures**

**
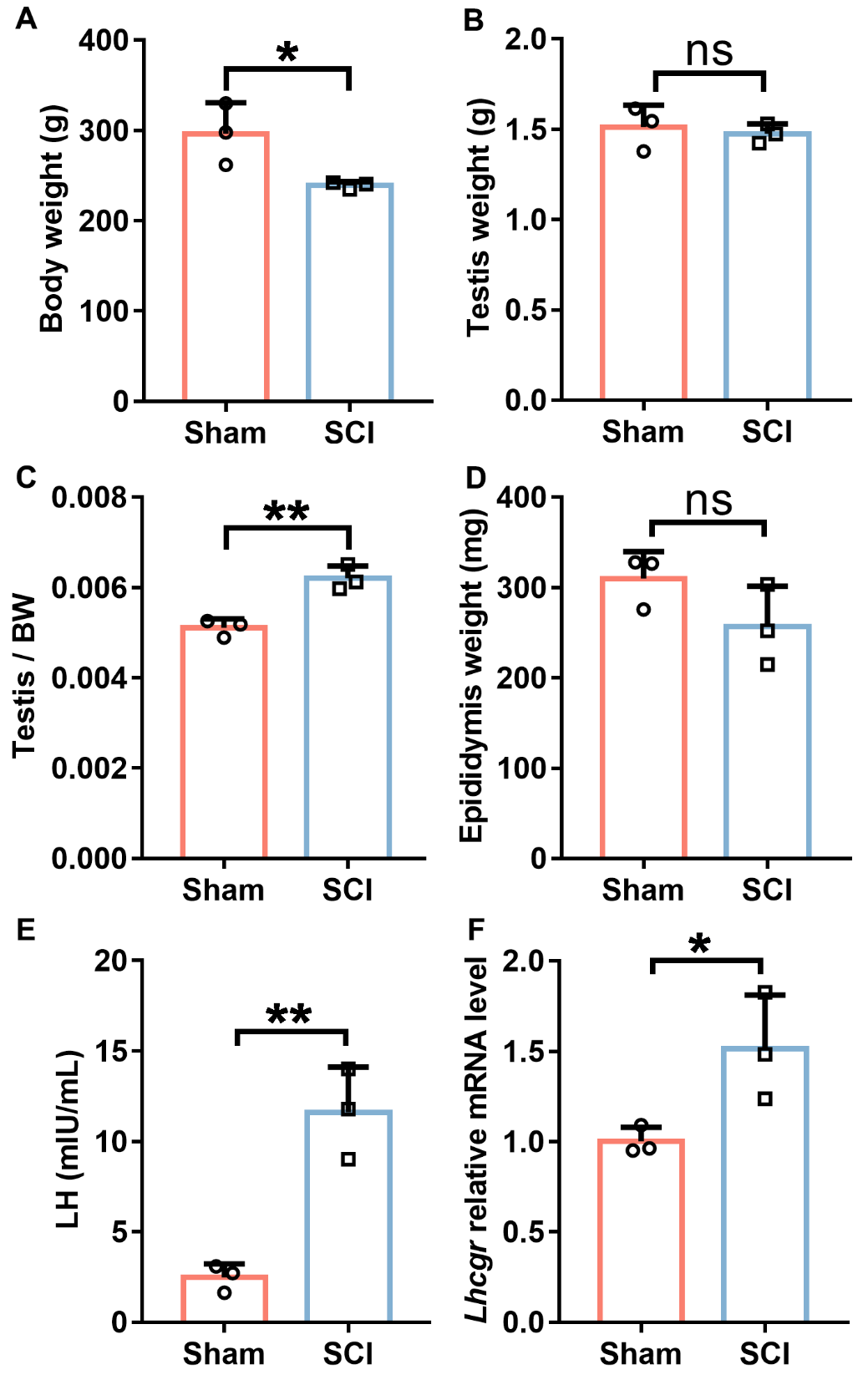
**

**Supplementary Figure S1** (A) Body weight of SCI and sham rats. (B) Testis weight of SCI and sham rats. (C) Ratio of testis/body weight between SCI and sham rats. (D) Epididymis weight of SCI and sham rats. (E) LH levels by ELISA assay. (F) qRT-PCR analyses of *Lhcgr* in testis of SCI and sham rats. Bars indicate mean values ± standard deviation. *n* = 3. ns = no significance; * *P* < 0.05; ** *P* < 0.01.

**
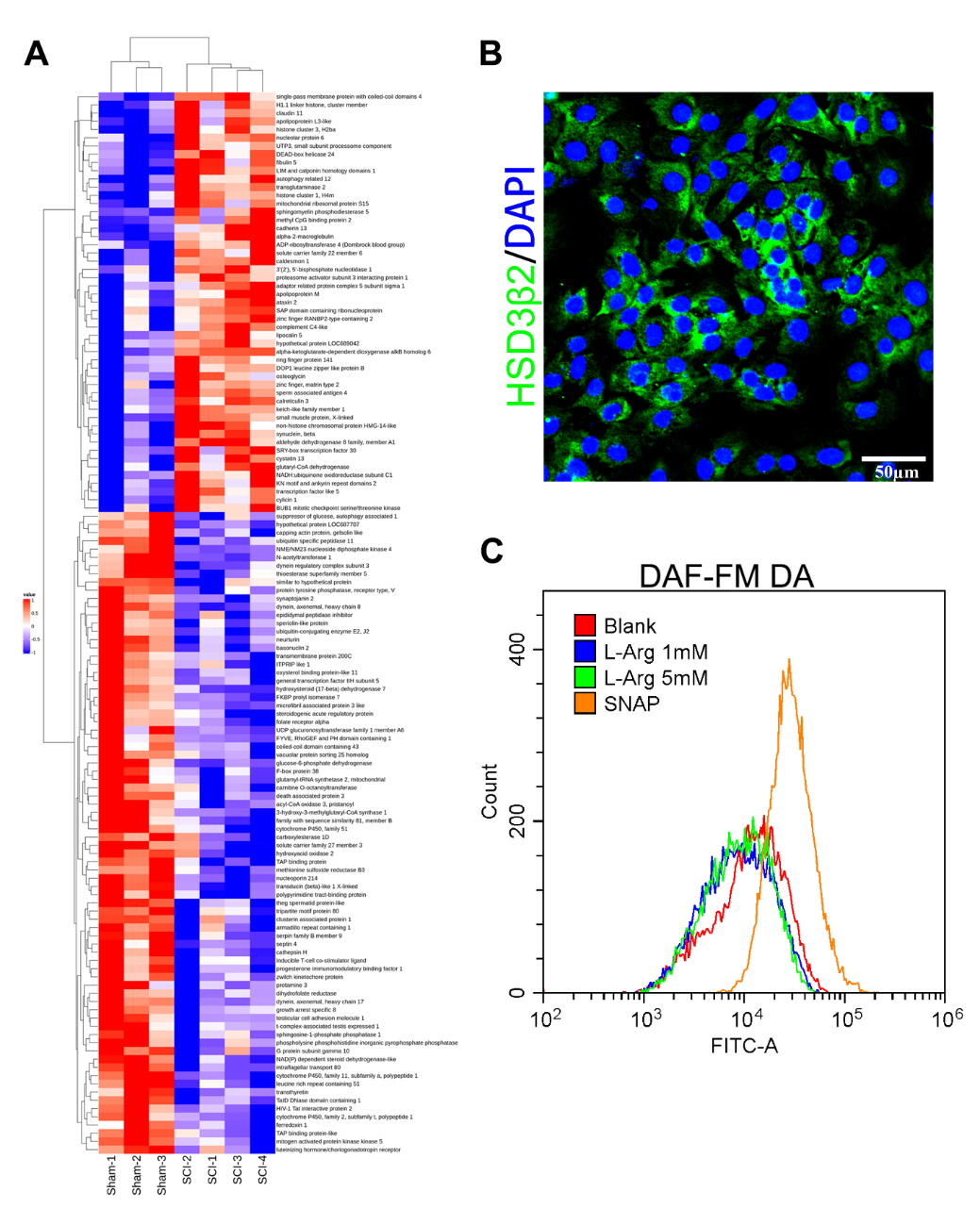
**

**Supplementary Figure S2** (A) Heat map of the 129 DEPs. Each column in the heat map represents a sample, and each row represents the expression level of a protein. (B) Identification of LCs by immunofluorescence of HSD3β2 (green). Nuclei were stained with DAPI (blue). Scale bar = 50 μm. (C) Cellular NO levels in L-Arg (1 mM, 5 mM)-treated and control LCs were detected by flow cytometry after DAF-FM DA (green) probing.

**
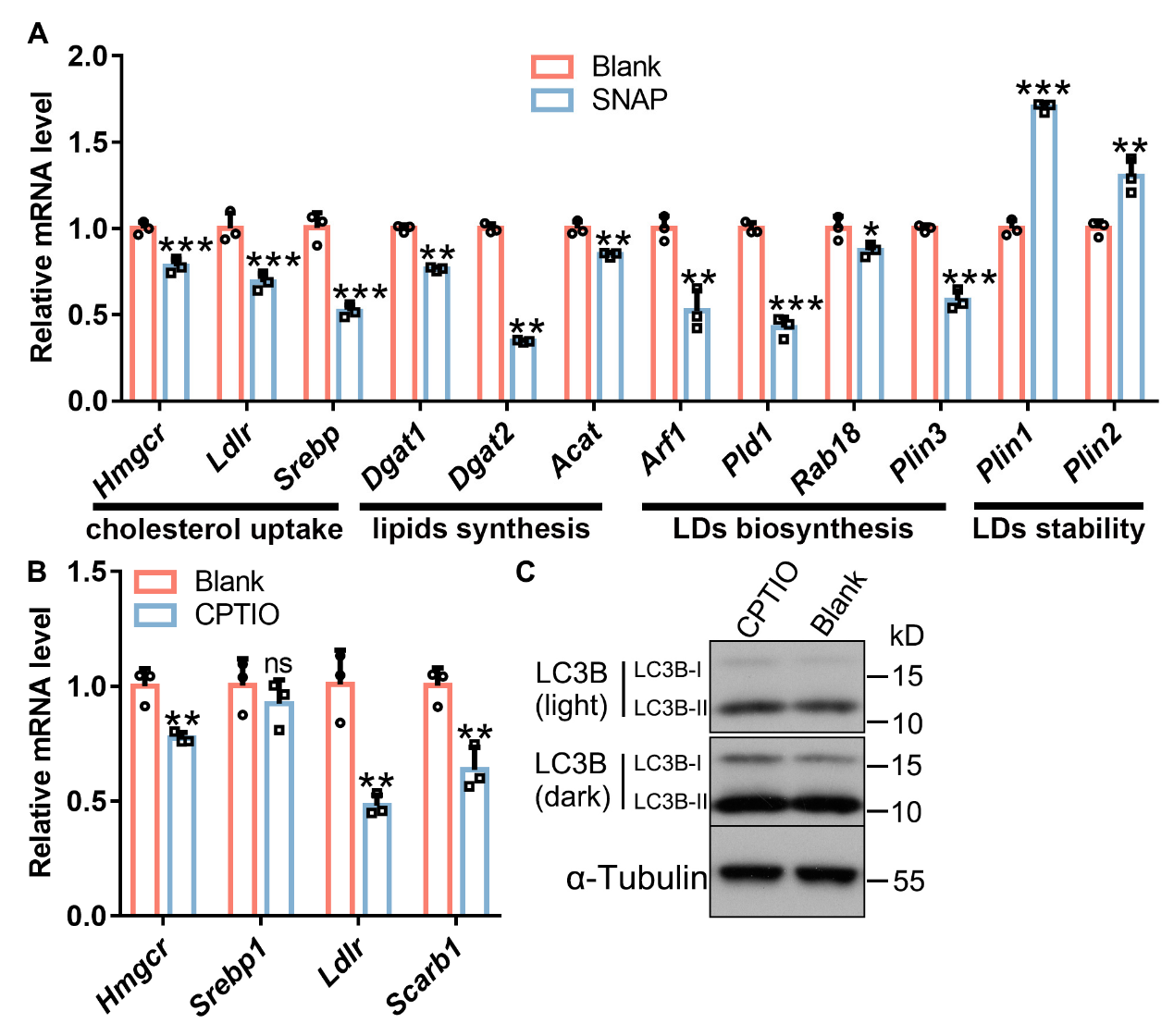
**

**Supplementary Figure S3** (A) qRT-PCR analyses of *Hmgcr*, *Ldlr*, *Srebp*, *Dgat1*, *Dgat2*, *Acat*, *Arf1*, *Pld1*, *Rab18*, *Plin3*, *Plin1* and *Plin2* mRNA levels in SNAP-treated and control LCs. (B) qRT-PCR analyses of *Hmgcr*, *Srebp1*, *Ldlr* and *Scarb1* mRNA levels in CPTIO-treated and control LCs. (C) LC3B-I/II immunoblotting in CPTIO-treated and control LCs. Bars indicate mean values ± standard deviation. *n* = 3. ns = no significance; * *P* < 0.05; ** *P* < 0.01; and *** *P* < 0.001.

**
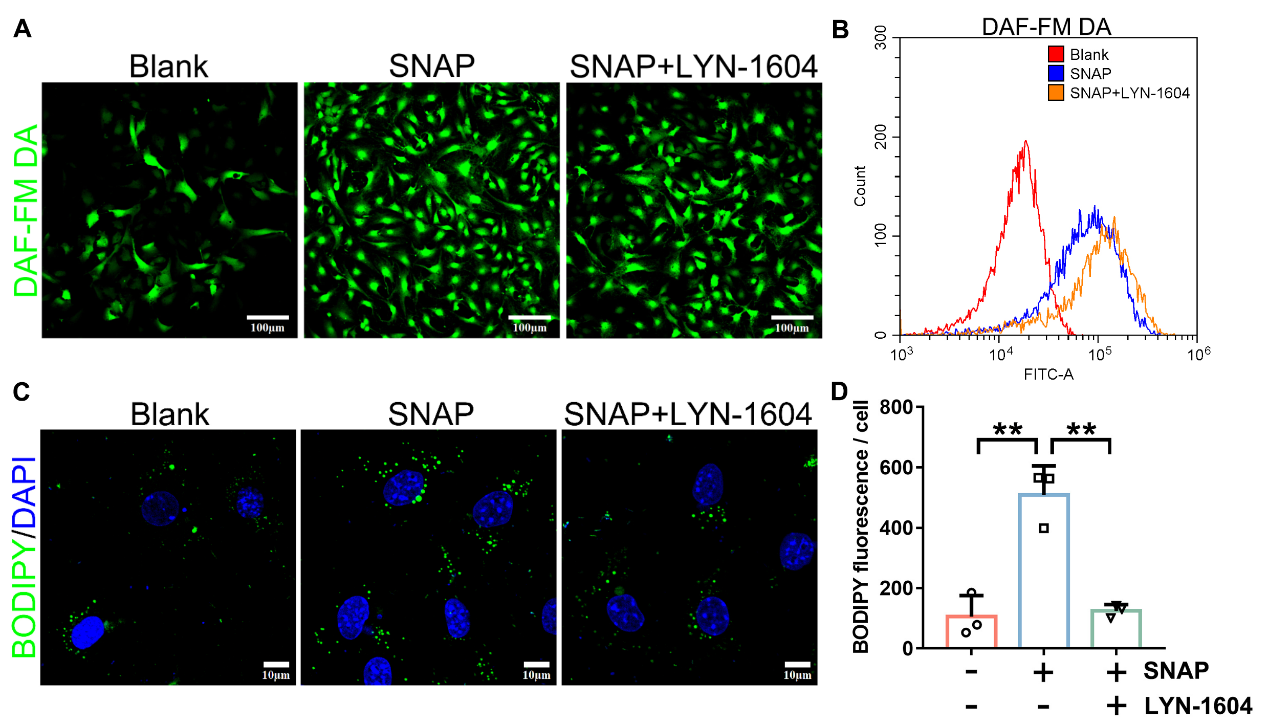
**

**Supplementary Figure S4** (A) DAF-FM DA fluorescence (green) showing cellular NO abundance in control, SNAP-, and SNAP+LYN-1604-treated primary LCs. LCs were cultured in normal medium, or in medium plus SNAP (500 μM) alone or combined with LYN-1604 (50 μM) for 24 h. (B) Quantification of DAF-FM DA intensity by flow cytometry in (A). (C) BODIPY (green) staining shows LDs abundance in indicated groups. Nuclei were stained with DAPI (blue). (D) BODIPY fluorescence quantification in (A). Data indicate mean values ± standard deviation. *n* = 3. ** *P* < 0.01. Scale bar = 100 μm for (A) and 10 μm for (C).

**
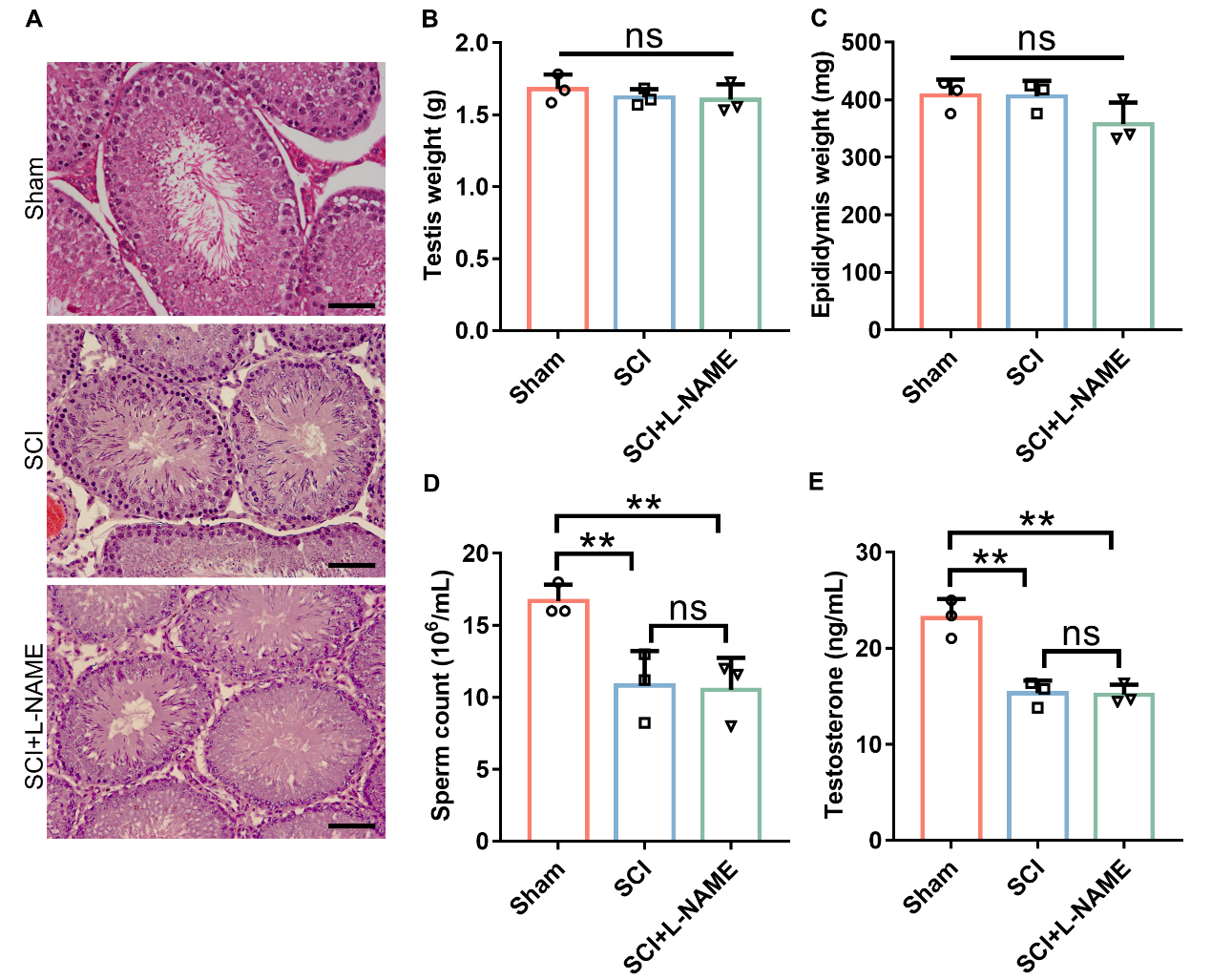
**

**Supplementary Figure S5. In vivo administration of L-NAME fails to restore spermatogenesis and testosterone level in SCI rats.** (A) H&E staining of testicular sections in sham, SCI and SCI+L-NAME groups. (B) Comparison of testis weight in indicated groups. (C) Comparison of epididymis weight in indicated groups. (D) Sperm number in indicated groups. Sperm were released from one epididymis from each rat. (E) Plasma testosterone level in indicated groups. Data indicate mean values ± standard deviation. *n* = 3. ns = no significance; * *P* < 0.05 and ** *P* < 0.01. Scale bar = 100 μm.

**
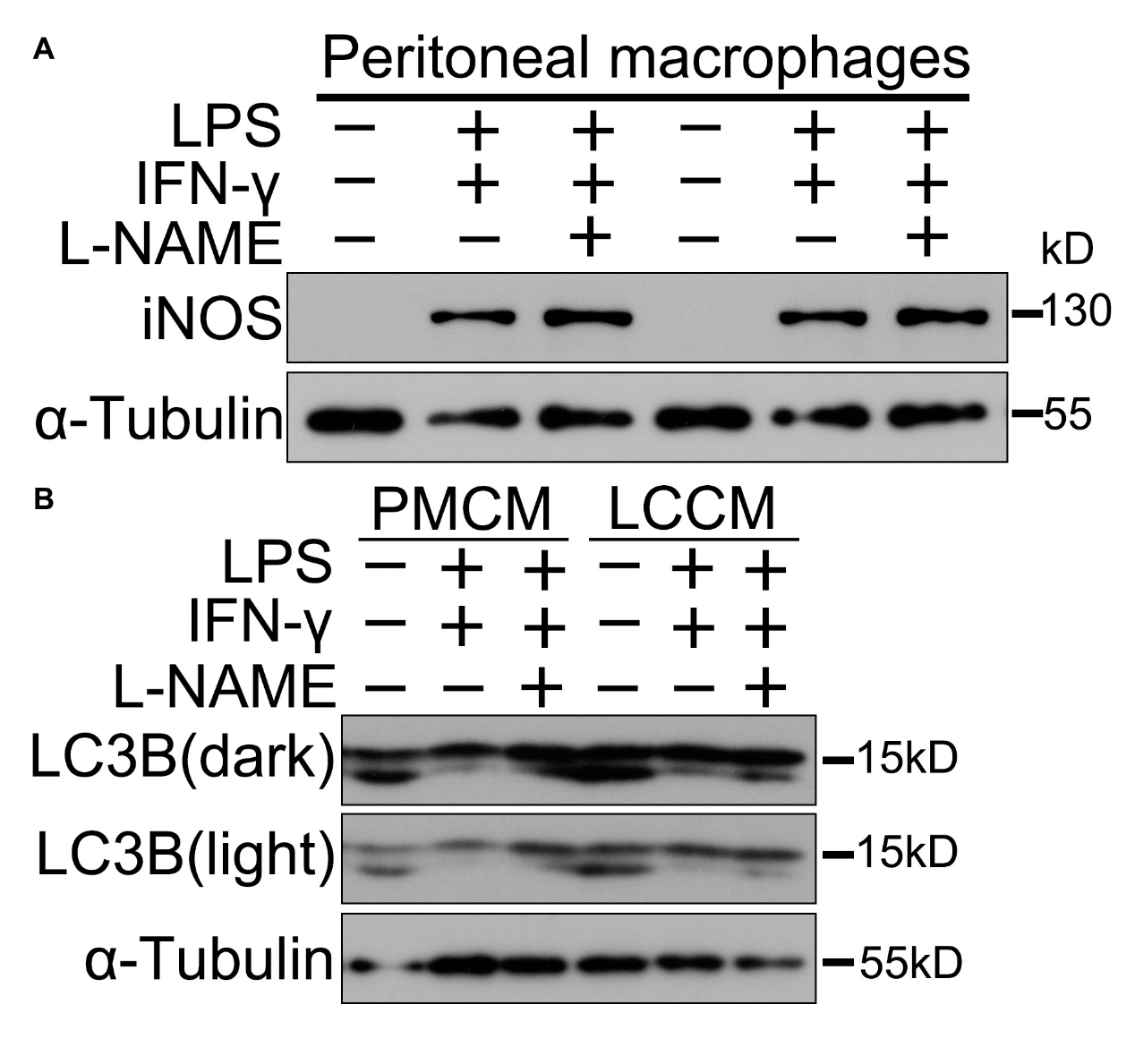
**

**Supplementary Figure S6** (A) iNOS immunoblotting in control, LPS+IFN-γ-, and LPS+IFN-γ+L-NAME-treated peritoneal macrophages. (B) LC3B-I/II immunoblotting in PMCM- and LCCM-treated LCs. PMCM, peritoneal macrophages culture medium. LCCM, Leydig cells culture medium.

**Supplemental Tables**

**Table S1. Antibodies.**

| **Antibody** | **Vendor** | **Catalog**  **number** | **Application** | **Working Dilution**  **for WB or (IF)** |
| --- | --- | --- | --- | --- |
| Phospho-mTOR (Ser2448) Rabbit Ab | Cell Signaling Technology | 2971 | WB | 1:1000 |
| mTOR Rabbit mAb | Cell Signaling Technology | 2983 | WB | 1:1000 |
| Phospho-S6 (Ser235/236) Rabbit Ab | Cell Signaling Technology | 2211 | WB | 1:2000 |
| S6 Mouse mAb | Cell Signaling Technology | 2317 | WB | 1:2000 |
| Phospho-ULK1 (Ser757) Rabbit mAb | Cell Signaling Technology | 14202 | WB | 1:1000 |
| Phospho-ULK1 (Ser555) Rabbit mAb | Cell Signaling Technology | 5869 | WB | 1:1000 |
| ULK1 Rabbit mAb | Cell Signaling Technology | 8054 | WB | 1:1000 |
| Phospho-AMPK(Thr172) Rabbit mAb | Cell Signaling Technology | 2535 | WB | 1:1000 |
| AMPKα (23A3) Rabbit mAb | Cell Signaling Technology | 2603 | WB | 1:1000 |
| SQSTM1/p62 Rabbit pAb | ZENBIO | 380612 | WB | 1:2000 |
| LC3B (E5Q2K) Mouse mAb | Cell Signaling Technology | 83506 | WB/IF | 1:1000 (1:200) |
| DDX4 (D10C5) Rabbit mAb | Cell Signaling Technology | 8761 | IF | (1:100) |
| HSD3β2 Rabbit pAb | ABclonal | A1823 | IF | (1:100) |
| CD68 Mouse mAb | Santa | 3F103 | IF | (1:100) |
| iNOS Rabbit mAb | Abcam | 178945 | IF | (1:100) |
| α-tubulin (MG17) Mouse mAb | Ray Antibody Biotech | RM2007 | WB | 1:4000 |

**Table S2. Primer sequences**

| Gene | Forward primer (5'→ 3') | Reverse primer(5'→ 3') |
| --- | --- | --- |
| *Gapdh* | AGGTCGGTGTGAACGGATTTG | TGTAGACCATGTAGTTGAGGTCA |
| *Star* | CGGGTGGATGGGTCAAGTTC | GCACTTCGTCCCCGTTCTC |
| *Lhcgr* | GAGACGCTTTATTCTGCCATC | CACCCTAAGGAAGGCATAGC |
| *Cyp11a1* | CACAGACGCATCAAGCAGCAAAA | GCATTGATGAACCGCTGGGC |
| *Hsd3b1* | TGGACAAAGTATTCCGACCAGA | GGCACACTTGCTTGAACACAG |
| *Hsd3b2* | CAG ACC ATC CTA GAT GTC | AGG A AG CTC ACA GTT TCC A |
| *Cyp17a1* | GCCCAAGTCAAAGACACCTAAT | GTACCCAGGCGAAGAGAATAGA |
| *Hsd17b3* | ATGGGCAGTGATTACCGGAG | ACAACATTGAGTCCATGTCTGG |
| *Hsd3b6* | ATCCACACTGCAGCTGTCATTG | ATGATGCTCTTCCTCGTTGC |
| *Dgat1* | TCCGTCCAGGGTGGTAGTG | TGAACAAAGAATCTTGCAGACGA |
| *Dgat2* | GCGCTACTTCCGAGACTT | GGGCCTTATGCCAGGAAACT |
| *Hmgcr* | CTGGAATTATGAGTGCCCCAAA | ACGACTGTACTGAAGACAAAGC |
| *Ldlr* | AGTGGCCCCGAATCATTGAC | CTAACTAAACACCAGACAGAGGC |
| *Acat* | CAGGAAGTAAGATGCCTGGAAC | TTCACCCCCTTGGATGACATT |
| *Srebp* | CAGTGGGGATGCACTTGGAAT | GAGTAACCCTTCACAGGCGT |
| *Arf1* | TGGGCGAAATTGTGACCACC | TCCACTACGAAGATCAAGCCT |
| *Pld1* | TCAAGCGACAGCACACACGAG | TGTTCTCAGTCCCAAACGA |
| *Rab18* | TTTGCACGCAAGCATTCTATGT | TTGTTCTGGTTCTCACTTTCCC |
| *Plin1* | GGGACCTGTGAGTGCTTCC | GTATTGAAGAGCCGGGATCTTTT |
| *Plin2* | GACCTTGTGTCCTCCGCTTAT | CAACCGCAATTTGTGGCTC |
| *Plin3* | ATGTCTAGCAATGGTACAGATGC | CGTGGAACTGATAAGAGGCAGG |
